# Antiviral Activity and Mechanisms of Seaweeds Bioactive Compounds on Enveloped Viruses—A Review

**DOI:** 10.3390/md20060385

**Published:** 2022-06-08

**Authors:** Silvia Lomartire, Ana M. M. Gonçalves

**Affiliations:** 1 University of Coimbra, Faculty of Sciences and Technology, MARE-Marine and Environmental Sciences Centre, Department of Life Sciences, Calçada Martim de Freitas, 3000-456 Coimbra, Portugal; silvia.lomartire@student.uc.pt; 2 Department of Biology and CESAM, University of Aveiro, 3810-193 Aveiro, Portugal

**Keywords:** seaweed, polysaccharide, polyphenol, antiviral activity, enveloped virus, IAV, HIV, HSV

## Abstract

In the last decades, the interest in seaweed has significantly increased. Bioactive compounds from seaweed’s currently receive major attention from pharmaceutical companies as they express several interesting biological activities which are beneficial for humans. The structural diversity of seaweed metabolites provides diverse biological activities which are expressed through diverse mechanisms of actions. This review mainly focuses on the antiviral activity of seaweed’s extracts, highlighting the mechanisms of actions of some seaweed molecules against infection caused by different types of enveloped viruses: influenza, *Lentivirus* (HIV-1), Herpes viruses, and coronaviruses. Seaweed metabolites with antiviral properties can act trough different pathways by increasing the host’s defense system or through targeting and blocking virus replication before it enters host cells. Several studies have already established the large antiviral spectrum of seaweed’s bioactive compounds. Throughout this review, antiviral mechanisms and medical applications of seaweed’s bioactive compounds are analyzed, suggesting seaweed’s potential source of antiviral compounds for the formulation of novel and natural antiviral drugs.

## 1. Introduction

Viral infections are common and recurrent across the globe, and the lack of effective treatments for diseases of viral origin puts a serious burden on public healthcare systems. Virology and pharmaceutics are focused on the development of broad-spectrum antiviral drugs, which are effective, present low toxicity, and are inexpensive. The first class of antiviral agents are identified with nucleosides, synthetic drugs that act faster and provide the maximum therapeutic effect. However, the side-effects associated with these drugs could lead to health issues, such as acute renal failure, as the drug doses increase, as well as addiction, with a consequential absence of the desired effect in the future [1]. To reduce the collateral effects, new and effective antiviral agents are therefore urgently required. In the last decades, the activity of herbal antiviral drugs with a wide spectrum of action for viruses has been reported. Natural drugs developed with natural compounds have been shown to help alleviate the symptoms of specific viral diseases and shorten the disease period, are less toxic, and the side effects are minimized [2]. The development of an effective antiviral agent depends on the type of virus to defeat, its mechanisms of infection and replication. Viruses are classified as simple and complex; simple (or non-enveloped) viruses are composed of a nucleic acid and protein envelope (capsid); complex (or enveloped) viruses are surrounded by a lipoprotein envelope (supercapsid) over the capsid, which makes them more vulnerable to adverse environmental factors [3]. The presence/absence of the envelope may condition their susceptibility to different disinfectants or antiviral agents.

Generally, the virus mechanism of infection includes three steps: (1) the virus is absorbed by the host cell through a specific interaction between surface proteins of the virus and the receptors located on the surface of host cells. The virus undergoes a modification and releases its internal structure into a state which will cause infection; (2) during the second phase, complex processes allow the expression of the viral genome to replication; (3) in the third and last step, the viral offspring obtained is released out of the host cell by the budding of lysis, going on to infect other host cells. Currently, pharmacology is researching and developing novel antiviral agents that are able to block the virus entry or interfere at each stage of virus replication, reducing the incubation time and disease [4,5,6,7,8].

In the present work, seaweed compounds with antiviral properties are discussed, as they could have potential as a prophylactic agent and could be involved in the development of natural therapeutic agents for patients with viral infections.

The mechanisms of action of seaweed’s bioactive compounds against enveloped viruses such as influenza, *Lentivirus*, Herpes viruses and coronaviruses are analysed throughout the manuscript. Seaweed-derived antiviral drugs possess a broad-spectrum against viral infection, they are less toxic or non-toxic at all, thus the side effects are not as harmful as for synthetic compounds. The aim of the present review is to show the importance of seaweed’s’ compounds in the pharmaceutical field, with a particular focus on virology.

## 2. Mechanisms of Infection for Enveloped Viruses

To investigate the antiviral mechanism of action of seaweed’s bioactive compounds it is important to acknowledge the mechanism of infection of interested viruses. In the following sections are described cycles of infection of the influenza virus, *Lentivirus*, Herpes viruses and coronaviruses, to make the reader understand the antiviral pathway performed by seaweed bioactive compounds against enveloped viruses.

### 2.1. Influenza Viruses

Influenza A virus (*Alphainfluenzavirus*) and Influenza B virus (*Betainfluenzavirus*) both belong both to fam. Orthomyxoviridae, composed by RNA viruses.

Influenza epidemics are mainly caused by Influenza A, the most common influenza infection during the flu season, which causes mild to severe illness and affects humans and animals [9,10], and type B influenza infection, which is highly contagious and can sometimes cause serious illness. Influenza B virus is reported to be less common during flu season and causes minor localized outbreaks [11,12]. Influenza A and B viruses symptoms include cough, sore throat, nasal discharge, fever, headache, and muscle pain. However, the symptoms can be more severe and lead to serious complications like bronchitis and pneumonia [13].

Influenza C viruses primarily infect humans and cause illness in some animals, such as swine. It causes mild upper respiratory symptoms, and is found in minor localized outbreaks [12,14]. The Type D viruses mainly affect pigs and cattle and are not known to cause humans infection [12,15].

**Figure 1:**
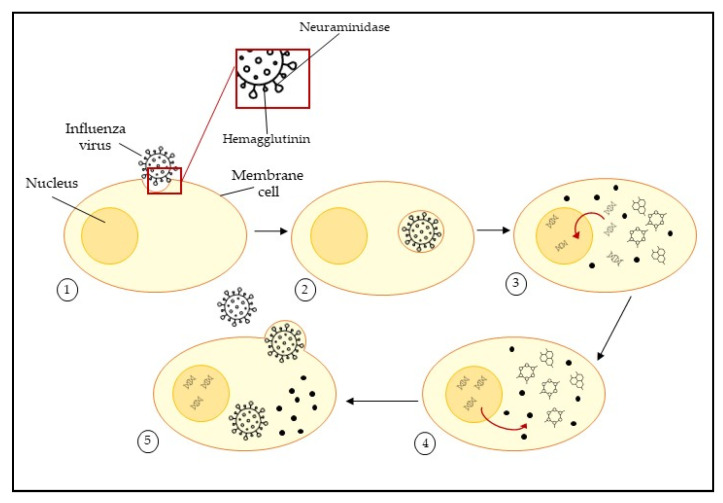
Schematic representation of influenza virus mechanism of cell infection. ① the spike protein hemagglutinin bond with the receptor on the host cell membrane; ② the virus enters into the cell by endocytosis; ③ the spike protein neuraminidase mediates the viral RNA and release, which enters the nucleus where it is replicated by the viral RNA polymerase; ④ viral mRNA is used to make viral proteins; ⑤ new viral particles are released into the extracellular matrix and the host cell continues to make new virus particles.

Influenza viruses are enveloped viruses that possess surface glycoproteins, mainly hemagglutinin (HA) and neuraminidase (NA). These glycoproteins play a key role in cycle infection (Figure 1); hemagglutinin mediates cell attachment and infection trough release of the viral genome into the cell, while neuraminidase mediates the release of progeny virions. These two protein spikes are very exposed; therefore, they are ideal targets for the development of vaccines, antibodies, and antiviral drugs.

Hemagglutinin inhibitors act during the first infection phase, preventing the attachment of the glycoprotein to target cells. Neuraminidase inhibitors prevent the release of the virus progeny into host cells.

The HA bind to sialic acids on the surface of epithelial cells of the respiratory tract, dendritic cells, type II pneumocytes, alveolar macrophages, or retinal epithelial cells [16,17]. Viruses enter by endocytosis, and follow the degradation of M1 and M2 proteins and release vRNPs, which transcribe viral mRNAs for the production of viral proteins in the cytoplasm and successively it enters the nucleus [18,19,20]. In the nucleus, negative-sense vRNA is transcribed into positive-sense mRNA using viral polymerase [21]. The viral proteins are translated from mRNA in the cytoplasm by ribosomes. IAVs complete successful replication by relying on multiple cellular proteins. Cellular clathrin, epsin-1 Ras-related GTPases, and COPI are important proteins for virus dynamin-dependent endocytic uptake. They degrade the M1 shell and uncoat vRNPs. Subsequently, cytoplasmic importins mediate the nuclear import of vRNPs through the nuclear pore complex (NPC). In the cytoplasm, a translation apparatus translates viral mRNAs into proteins and GRSF1 stimulates this process. Newly synthesized M1, M2, HA, and NA are also transported to the plasma membrane through the trans-Golgi network with the help of COPI and Rab8. β-actin, CK2 and Rab11 are cellular proteins required for the budding and release of new virions [13].

Early studies on HA revealed that HA-mediated fusion involves the insertion of a key segment into the host membrane and that segment is known as the influenza fusion peptide (IFP). It has been shown that mutations within the FP region of HA can either maintain its ability to induce complete fusion or only promote hemifusion (fusion of the outer leaflets of two membranes), depending on the residue which is mutated [22]. Therefore, more studies focusing on characterizing its structure and effect on membranes of host cells can help to find new anti-influenza treatments that block the fusion of the virus into the host cell [23].

Mutation in amino acids in IAV proteins can cause antigenic drift, which allows emerging viruses to evade host immunity developed from previous IAV infections or vaccinations. One of the causes of mutation can be due to the error-prone nature of viral polymerase [24]. The viruses can also undergo rearrangement of genetic segments to generate different variations and sometimes antigenic shift. Genetic shifts and drifts are potential causes of epidemic and pandemic outbreaks, and are sometimes even dangerous [25].

Among antivirals, oseltamivir represents the most used agent against influenza viruses, which inhibit neuraminidase action. Oseltamivir proved potential clinical utility against seasonal and emerging influenza viruses [26].

### 2.2. Lentivirus

HIV belongs to the genus *Lentivirus*, fam. Retroviridae. It is an RNA enveloped virus with an unusual method of replication of genetic material. Lentiviruses can cause diseases with a long incubation period and a slow but steady progressive course that involves the central nervous system [27]. HIVs are usually grouped into two types: HIV-type 1 (HIV-1), also known as the main acquired immunodeficiency syndrome (AIDS) agent, and HIV-type 2 (HIV-2), present mainly in some regions of Western and Central Africa [28]. The structure of *Lentivirus* is made of the retrovirus genome, composed of two identical copies of single-stranded RNA molecules, major proteins such as glycoproteins, group antigens polyproteins (GAg), reverse transcriptase (pol), and envelope proteins (Env). Reverse transcriptase is the essential enzyme that carries out the reverse transcription process to produce molecules of complementary double-stranded DNA pre-formed from viral RNA. Additionally, HIV-1 and HIV-2 viruses possess other regulatory genes which can cause immunodeficiency disorders such as AIDS. It appears that AIDS is more frequent in HIV-2 infection [29], but it appears less virulent than HIV-1 and the infection course takes longer to develop into AIDS compared to HIV-1 [30,31,32].

**Figure 2:**
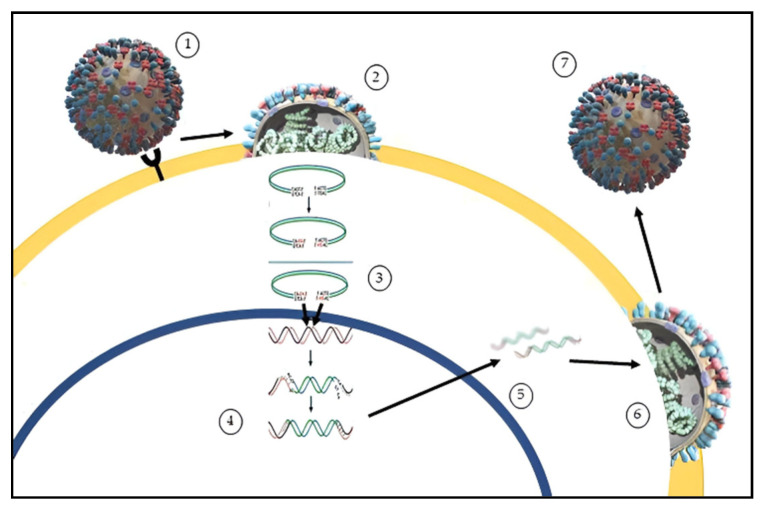
Schematic representation of HIV infection. ① binding of the virus to the host cell membrane; ② fusion of the virus and uncoating: the virus core uncoats into the cytoplasm of the target cell freeing the viral RNA; ③ reverse transcription: the viral RNA is transcribed into an RNA/DNA hybrid double helix; ④ integration of the viral gene to human DNA; ⑤ replication; ⑥ assembly of the new viral particles and proteins; ⑦ budding process and release of the new virus.

The HIV replication cycle can be summarised in six steps (Figure 2): (1) binding and entry, HIV-1 and HIV-2 possess heterodimer proteins gp120 and gp41 that compose the viral envelope, which are essential for virus recognition and entry into target cellular membranes; (2) uncoating, the virus core uncoats into the cytoplasm of the target cell freeing the viral RNA; (3) reverse transcription, the viral RNA is transcribed into an RNA/DNA hybrid double helix. The ribonuclease enzyme breaks down the RNA strand and the polymerase active site of the reverse transcriptase completes a complementary DNA strand to form a double helix DNA molecule which is integrated within the cell genome through integrase enzyme; (4) provirus integration, where the viral messenger RNA coding for long fragments migrates into the cytoplasm, where (5) new virus proteins are synthesized, such as GAg, pol, Env. The formation of new HIV virus derives from the association of two viral RNA strands incorporated together with replication enzymes, while core proteins assemble over them, forming the virus capsid. This immature and infective particle migrates towards the cell surface, where they bud through the host cell membrane, acquiring a new envelope. During the last step, the (6) budding process, the virus lipid membranes may incorporate various host cell proteins and become enriched with phospholipids and cholesterol and the virus goes to infect other cells [28].

HIV attachment and fusion with the cell membrane is mediated by the viral glycoprotein (Env) and the CD4 receptor and coreceptor (CXCR4 or CCR5) [33]. The viral capsid containing the HIV-1 genome and replicative enzymes is then released into the cytoplasm where the viral reverse transcriptase (RT) enzyme transcribes the viral RNA genome into a double-stranded DNA copy.

In the nucleus, the viral integrase (IN) enzyme directs the integration of the viral DNA into transcriptionally active sites within the host chromatin. The integrated proviral DNA is then transcribed into viral mRNAs and full-length genomic RNA, and the GAg and Pol polyproteins are targeted to the inner leaflet of the plasma membrane where they assemble into an immature hexameric lattice. The Env glycoprotein precursor, gp160, is cleaved by host furin or furin-like proteases to generate the surface glycoprotein subunit gp120 and the transmembrane glycoprotein gp41. Viral genomic RNA dimers are packaged by the assembling Gag lattice and heterotrimeric gp120/gp41 Env complexes are incorporated into the membrane at the sites of assembly [34]. Immature Pol polyprotein are arranged into mature proteins by the viral protease (PR). PR mediated Gag cleavage triggers the disassembly of the membrane-bound immature Gag lattice, resulting in the assembly of the mature conical capsid from the fully processed capsid protein [35]. The virion assumes its proper infectivity when it reaches maturation that requires the packaging of two single-stranded copies of the viral RNA genome, together with RT and IN, into the nascent capsid and then is ready to bud and infect the organism [36].

### 2.3. Herpes Viruses

Herpes viruses (fam. *Herpesviridae*) are extremely successful parasites developed for millions of years, evolving action mechanisms to coexist with their hosts and to maintain host-to-host transmission and lifelong infection by regulating their life cycles. Human Herpes viruses are the causative agents of many common diseases, including chickenpox, shingles, mononucleosis, cold sores, and genital Herpes [37]. Eight Herpes viruses are known pathogens of humans: α-herpesviruses (Herpes simplex virus-HSV type 1 and type 2, Varicella zoster virus -VZV), cytomegalovirus, β-herpesviruses (Human Herpes virus-HHV 6 and 7) and γ-herpesviruses (Epstein–Barr virus, HHV8) [38].

Herpes viruses have a unique four-layered structure: a core containing the double-stranded DNA genome, which is enclosed by an icosapentahedral capsid composed of capsomers. The capsid is surrounded by an amorphous protein coat called the tegument. It is encased in a glycoprotein-bearing lipid bilayer envelope.

After the entry into the host cell, the life cycle of HSVs consists of two phases: lytic infection and latent infection [39,40,41,42,43]. During lytic infection, virions into host cell progress through uncoating, gene transcription, DNA replication, protein translation, assembly, release, etc., to produce progeny virions [43,44,45]. During this stage, the replication of HSVs is limited by the immune system, leading to the stage of latent infection.

HSV-1 and HSV-2 contain a large, linear double stranded DNA genome protected by an icosahedral capsid surrounded by a proteinaceous layer termed the tegument and are wrapped in an envelope containing viral glycoproteins. Initial attachment to the plasma membrane occurs through the binding of glycoprotein B (gB) and gC to glycosaminoglycans (GAG), attached to the outer surface of the cell membrane [46]. The interaction of HSV-1 gH/gL with specific integrins leads to HSV-1 entry through endocytosis [47]. Fusion can take place at the plasma membrane or within vesicles following viral internalization. Following fusion, some tegument proteins, like VP16, dissociate from the capsid and travel to the nucleus independently [48], while others remain bound. Inner tegument proteins mediate interaction with dynein, dynactin and kinesin motor proteins and facilitate capsid transport on microtubules toward the nucleus. The viral linear DNA genome enters the nucleus through a nuclear pore [49], and the RNA polymerase II and viral proteins transcribe HSV genes. Gene expression follows an ordered cascade during lytic replication. Immediate early genes are expressed in the absence of de novo viral protein synthesis. The tegument protein VP16 forms a complex with host cell factor 1 (HCF-1) and octamer binding protein-1 (Oct-1) that binds to the promoter of ICP, driving their expression [50]. Once DNA replication occurs, late genes which involved virus assembly are expressed. Viral transcription, DNA replication, capsid assembly and DNA encapsulation occur exclusively in the nucleus. Mature capsids containing viral DNA, leave the nucleus through a process mediated by pUL31 and pUL34. Following exit from the nucleus, cytosolic capsids acquire more inner tegument proteins, while outer tegument proteins and viral membrane proteins are incorporated at the membrane compartments of trans Golgi network vesicles and endosomes [51,52].

The mechanisms leading to transport and incorporation of viral glycoproteins are not completely understood. Data showed that pUL36 and pUL37 mediate motility in the neuronal cell body but cannot direct the non-enveloped capsids to the axons, contrary to vesicles containing gD that efficiently employed axonal transport [53]. These results suggest that only fully assembled viral particles can travel from the cell body to the axon termini. Vesicles transport HSV particles to the plasma membrane and enveloped HSV exits the cell upon fusion of the vesicle with the plasma membrane [54].

During latent infection, the viral genome remains in an inactive state in the host cell, but it can re-start the lytic cycle under immune suppression or environmental stimuli [55,56]. HSVs start the active replication to produce large numbers of infectious virions that can be transmitted to new hosts [57]. For example, HSV latency has been detected in neurons. Infection of susceptible non-neuronal cells normally leads to lytic replication, although a recent report suggested the existence of latency in a proportion of non-neuronal cells [58], even though latency mechanisms in neurons are not yet completely clear [59,60,61].

### 2.4. Coronaviruses

Human coronaviruses such as HCoV-229E and HCoV-OC43 have long been known to circulate in the population; all strains are identified to cause seasonal and usually mild respiratory tract infections associated with symptoms of the ‘common cold’. Over the last years, Severe Acute Respiratory Syndrome Coronavirus (SARS-CoV), Middle East Respiratory Syndrome Coronavirus (MERS-CoV) and SARS-CoV-2 have emerged in the human population. These strains are highly pathogenic and can infecting bronchial epithelial cells, pneumocytes and upper respiratory tract cells in humans, developing severe life-threatening respiratory pathologies and lung injuries. Infected patients with SARS-CoV-2 reported a high rate of morbidity and elevated mortality, making this new coronavirus one of the most important threats to humankind in the last few centuries [62].

Coronaviruses are mainly composed of four main structural proteins: spike (S), envelope (E), membrane (M), and nucleocapsid (N) [63].

S protein is composed of two functional subunits, S1 and S2. S1 binds with the receptor on host cell, while the S2 subunit fuses the membranes of viruses and host cells [64]. The positive-sense, single-stranded RNA viral genome is protected by nucleocapsid proteins, whereas membrane proteins and envelope proteins ensure its incorporation in the viral particle during the assembly process. Coronavirus infection involves the specific binding of the spike protein to the cellular entry receptors, which have been identified for several coronaviruses and include human aminopeptidase N (APN) for HCoV-229E, angiotensin-converting enzyme 2 (ACE2) for HCoV-NL63, SARS-CoV and SARS-CoV-2, and dipeptidyl peptidase 4 (DPP4), for MERS-CoV. The interaction between spike protein and cell receptor is aided by host factors, such as the cell surface serine protease TMPRSS2, which promotes the viral uptake and fusion at the cellular or endosomal membrane. Viral RNA is released and uncoated and then translates into two large open reading frames, ORF1a and ORF1b, that encode polyproteins pp1a and pp1ab involved in the viral replication and transcription complex. Translated structural proteins translocate into endoplasmic reticulum membranes and transit through the ER-to-Golgi intermediate compartment, where interaction with N-encapsidated, newly produced genomic RNA results in budding into the lumen of secretory vesicular compartments. Finally, virions are secreted from the infected cell by exocytosis [63]. The route of transmission for SARS-CoV-2 is primarily through respiratory droplets, aerosol, direct contact with contaminated surfaces, and faecal–oral transmission [65,66,67].

The adaptative mutations in the SARS-CoV-2 genome could alter its pathogenic potential, thus the difficulty of finding drugs or vaccines to block the transmission of the virus increases. Therefore, it is important to obtain further information regarding the mechanisms of action of SARS-CoV-2 in order to find treatments to reduce symptoms and reduce mortality rates [62].

## 3. Antiviral Potential of Seaweed Bioactive Compounds

The scientific progress of the last decades allows the development of new research methods and tools for the investigation of marine resources as potential sources of biological compounds useful for pharmacological applications.

Seaweeds have been widely explored for their powerful properties, which include antiviral activity. Each compound expresses a diverse antiviral response, depending on structure, molecular weight, and mechanism of action. Among the compounds investigated, sulphated polysaccharides and polyphenols show higher antiviral activity.

The general mechanism of action of sulphated polysaccharides against virus appears to be based on the interference of the initial attachment of the viral external glycoprotein and the negatively charged constituents of the host cell surface. Sulphated polysaccharides present a high density of negative charge in their molecule; therefore, they can interact with the positively charged constituent of the viral glycoproteins, blocking the entry of the virus into the target cell [68,69,70]. Among sulphated polysaccharides, fucoidans demonstrated antiviral properties against different viral strains, for example human immunodeficiency virus (HIV), Herpes simplex virus (HSV), human cytomegalovirus (HCMV), influenza virus (IAV) and bovine viral diarrhoea virus (BVDV) [69,71,72,73,74].

Fucoidan polysaccharides are located in the cell wall as a cellular matrix component of brown algae. Their structures are made up of l-fucose backbone with other sugars often present alongside fucose, such as xylose, mannose, galactose, rhamnose, arabinose, glucose, glucuronic acid and acetyl groups, depending on the variety of brown algae [75]. Fucoidans mainly inhibit the virus attachment to host cells by interfering with the external viral glycoproteins involved in target-cell attachment [76,77], while a study by Sun et al. [78] reported an increase of immune system response after fucoidan absorption. Low-molecular weight from *Laminaria japonica* were tested in vitro and in vivo against Adenovirus and Parainfluenza virus. The results indicated a remarkable antiviral activity in vitro for two types of fucoidans at high dose, while in vivo they could prolong the survival time of virus-infected mice and fucoidans improved the action of the immune system by increasing thymus and spleen indices, which reflect the strength of body’s innate immune function [79]. Some antiviral assays revealed that polysaccharides can inhibit the virus transcription and replication process in host cells. Fucans isolated from *Fucus vesiculosus* appear to have an evident inhibitory effect on HIV reverse transcriptase (RT) at a concentration of 0.5–1.0 mg/mL. Fucans have specific sugar rings that spatially orientate and change configuration to recognize the enzyme and determine the binding to inhibit the transcription of viral DNA [80].

Laminarin, a water-soluble polysaccharide extract from brown algae, also reported antiviral activity against HIV [81]. Its structure is based on glucose units composed of (1,3)-β-D-glucan with β-(1,6)-branching. Laminarin from kelp inhibited the adsorption of HIV on lymphocytes and the activity of HIV reverse transcriptase at the concentration of 50 μg/mL, which suggests that laminarin polysaccharides are a good constituent for HIV treatments [82].

Alginate is a linear polymeric acid extracted from brown algae widely used for industrial applications. It has also been explored for its antiviral properties [83,84,85,86]. The alginate structure is composed of 1,4-linked β-D-mannuronic acid and α-L-guluronic acid residues, which have different configurations [87,88]. Xin et al. [89] reported that anti-HIV marine drug 911 that contain alginate derivatives has inhibitory activity on the HIV reverse transcriptase in vitro and in vivo, and also interferes with the viral adsorption and improves the immune function of host cells. Moreover, alginate 911 can improve the immune function of host cells and inhibit the activity of hepatitis B virus (HBV) DNA polymerase, thus marine polysaccharides can also be used to inhibit the replication of HBV [90]. Fabra et al. [83] determined the antiviral activity of alginate incorporated in edible biofilm. The development of active films and coatings with antioxidant, antimicrobial, and antiviral properties is currently gaining interest, as they can lead to a lesser consumption of plastic for food packaging and may diminish the growth of pathogenic microorganisms affecting food quality and safety, due to the biological properties of seaweed incorporated. The antiviral activity of alginate-based film has been determined for murine norovirus (MNV-1) and hepatitis A virus (HAV). Virus suspensions incubated with alginate exhibit antiviral properties against the tested virus, suggesting that alginate might be exploited for the formulation of active films for food packaging or as constituents for antiviral drugs, even though further research must be addressed to better understand the antiviral activity of alginates [83].

Sulphated polysaccharides of red seaweeds are investigated for their antiviral properties [91,92,93]. Carrageenan is a hydrocolloidal polysaccharide most abundant in red algae cell walls, constituting from 30% to 75% of the algal dry weight [94]. Carrageenan is a linear polysaccharide formed from alternating sulphated or non-sulphated galactose units and linked with α-1,3-glycosidic bond and β-1,4-galactose bonds [95]. Depending on the position at which the ester sulphate group is connected on the galactose unit, carrageenan can be divided into different types (κ-carrageenan, ι-carrageenan, λ-carrageenan, γ-carrageenan, ν-carrageenan, ξ-carrageenan, and µ-carrageenan) based on solubility in potassium chloride. In nature, carrageenan are mostly hybrids, thus their properties vary based on the bonded sulphate group [96,97]. The antiviral effects of carrageenans are strictly dependent on the molecular weight and content of sulphated groups; indeed, the antiviral mechanism of actions are associated with different types of carrageenans, viruses and host cells [70,98,99,100].

Buck et al. [101] have demonstrated the antiviral action of different types of carrageenans against human papillomavirus (HPV) at the initial infective stage. The mechanism of action involves the direct binding of carrageenan to the viral capsid, and results proved that ι-carrageenan exhibits a higher antiviral response compared with λ- and κ-carrageenans, probably due to the ι-carrageenan sulphated polysaccharide sequences, which represent an ideal binding substrate for viral glycoproteins. ι-carrageenan is also reported to inhibit human rhinovirus (HRV) infection by interfering with the binding of viral glycoproteins and target cells [102]. Carrageenans, as well as fucoidans, exhibit antiviral activity and enhance the proliferation of lymphocytes, stimulating a rapid response of the immune system [103,104].

Polyphenolic compounds from terrestrial plants and seaweeds have been recently investigated for their antiviral properties, especially phlorotannins [105,106,107,108,109]. These polyphenols accumulated in brown seaweed are made up of monomeric units of phloroglucinol (1,3,5-hydroxybenzene) [110], and their heterogeneity and variations make them unique compounds able to exhibit different biological activities, including antiviral action [111].

It has been reported that phlorotannin can inhibit α-glucosidase, the enzyme involved in the polymerization of N-glycans. Most glycoproteins of enveloped viruses possess N-glycan chains, which are associated with glycoprotein maturation; consequently, the inhibition of α-glucosidase can prevent the development of the virus [112]. Therefore, phlorotannins have been proposed as broad-spectrum antiviral agents against enveloped viruses. Kim and Kwak [44], investigated the effect of phlorotannins from the brown alga *Eisenia bicyclis* on HPV, and results confirmed their antiviral activity against the two strains HPV-16PVs and HPV-18PVs [105]. Another example is given by Kwon et al. [113], which detect antiviral activity from phlorotannin by *Ecklonia cava* ethanol extract against the Porcine Epidemic Diarrhea Virus (PEDV). Among the extracted phlorotannins, phlorofucofuroeckol A and dieckol were the most abundant and recommended from the authors as potential agents to treat PEDV. The mechanism of action of polyphenols differs from the type of compounds and viruses involved. Park et al. [114] tested the antiviral activity of phlorotannin extract from *E. cava* as an inhibitor of SARS-CoV virus proteases. It was found that only specific phlorotannin can bind to viral protease and block the enzyme involved in the production of proteins necessary for the development of SARS-CoV virus [105].

## 4. Antiviral Properties of Seaweeds

In the following section, pre-clinical and clinical cases including seaweed’s compounds were investigated for their anti-influenza, anti-HIV and antiherpetic activity, as shown in (Table 1 and Table 2).

### 4.1. Anti-Influenza Activity of Seaweeds

Hayashi et al. [115], investigated fucoidans extracted from *Undaria pinnatifida* to verify the antiviral action against IAV in vivo and in vitro. The in vivo activity of fucoidans was evaluated in both immunocompetent and immunocompromised mice infected with IAV. Results showed that fucoidans inhibited both in vitro and in vivo replication of IAV; infected mice appeared to have stimulated and enhanced immune defences in both groups. In fact, an increase of antibody in bronchoalveolar lavage fluids of mice has been detected, likely due to the stimulation of the immune system. Immunocompromised mice treated with the antiviral oseltamivir were then submitted to prolonged viral replication, and from drug susceptibility tests it emerged that mice had less resistance to viruses. Further analysis confirmed that fucoidans resistant to IAV were not recovered from the immunocompromised mice, indicating that probably fucoidans might not interfere with viral replication within the host cell, but only act at the early stages of infection, interfering with the binding between virus and target cells. In immunocompromised mice, drug-resistant viruses often multiply after treatment with oseltamivir, while no resistant viruses were isolated from mice treated only with fucoidan. In light of this results, the authors proposed the combined treatment with oseltamivir and fucoidan. Under this combination there was no recurrence of influenza virus reproduction, as had happened in some cases when mice were treated only with oseltamivir. Moreover, after oral administration of fucoidans, results demonstrated their antiviral activity by reduced virus replication, weight loss, and mortality in animals of both groups, and increased their lifespan.

Mekabu fucoidan extracted from *Kjellmaniella crassifolia* was assayed for its antiviral potential against IAV in vitro multiplication. Madin-Darby Canine Kidney (MDCK) cells were infected with the virus and then treated with fucoidans. As the results showed, fucoidans significantly reduced the virus replication and promote cell viability. The plaque reduction assay was then performed to explore whether fucoidans directly inhibited the infection of viral particles before entering the host cell, and results proved the inhibition of viral infection on pre-incubated cells in presence of fucoidans. Results suggest that fucoidans may be able to inactivate viral particles and act like other neuraminidase inhibitors [116].

The immunomodulatory effect of mekabu fucoidan after influenza virus infection has also been investigate by Negishi et al. [165]. The authors investigated antibody production after influenza vaccination in two groups of elderly Japanese men and women, one group under oral fucoidan intake and one placebo group. The fucoidan-intake group had higher antibody titers against all three strains contained in the seasonal influenza virus vaccine than the placebo group. In the treated group, natural killer cell activity tended to increase after fucoidan intake, while in the placebo group no substantial increase was noted. From these results, the authors suggest that intake of mekabu fucoidan from *U. pinnatifida* by the immunocompromised elderly might increase antibody production after vaccination, possibly preventing influenza epidemics [165].

Mekabu fucoidan on the viral replication and immune responses induced by avian influenza viruses (H5N3 and H7N2 subtypes) in mice was investigated by Synytsya et al. [127]. This polysaccharide presents a low molecular weight (9 kDa) fucogalactan, consisting of partially sulphated and acetylated fucose and galactose residues. The administration of *U. pinnafitida* polysaccharides produced during the two weeks after viral infection a dose-dependent higher antibody titre, and the level of virus replication also decreased. Oral administration of Mekabu fucoidan blocked the release of the virus from cells and significantly increased the titer of virus-neutralizing antibodies and IgA, showing favourable effects in the control of avian influenza virus infections.

A recent case published by Richards et al. [117] demonstrates the inhibition activity of fucoidan from *U. pinnatifida* in mice infected with severe influenza A (H1N1). Orally delivered fucoidan significantly reduced gross lung pathology due to severe H1N1 infection in an animal model when administered at the same time as the viral infection. When the fucoidan was included in a feed supplement three days prior to infection, it provided a significant level of protection against the clinical signs of influenza A, and gross lung pathology was reduced in a dose-dependent manner. The reduction in symptoms and lung consolidation in this model suggests the possibility to integrate fucoidan from the edible *U. pinnatifida* in nutritional supplements in the management of acute viral respiratory infection [117].

Brown seaweed is also a great source of polyphenols. Several studies reported on the anti-influenza potentiality of polyphenols. Phlorotannins have been reported to interfere with viral proteins of IAV, specifically with neuraminidase. In the work of Ryu et al. [119], five phlorotannis were isolated from the ethanol extract of *Ecklonia cava*. The extract showed a strong anti-neuraminidase activity studied on various strains of influenza virus. The phlorotannin eckol showed a moderate IC_50_ value against the influenza A (H1N1) virus but was inactive towards other viral strains compared to the other compounds tested (7-phloreckol, phlorofucofuroeckol A, and dieckol). Spectral data showed the structure of phlorotannins and it appeared that with an increase in the number of hydroxyl groups (from eckol to dieckol), neuraminidase activity inhibition also increases [119]. All five phlorotannin derivatives were found to be selective inhibitors of neuraminidase activity, even though phlorofucofuroeckol A exhibits the strongest activity, suggesting its use for further development of anti-influenza drugs.

Cho et al. [120] recently investigated 13 phlorotannins extracted from *E. cava* and tested against influenza A viruses (strains H1N1 and H9N2). Results suggested that phlorofucofuroeckol A from *E. cava* plays a key role in the antiviral activities against H1N1 and H9N2 virus, as it has inhibitory effects on neuraminidase and hemagglutinin. The results showed six of the compounds with moderate to strong effects on both viruses, with the strongest antiviral activity for phlorofucofuroeckol A, confirming this phlorotannin as a potential agent for the further development of anti-influenza drugs [120].

The antiviral potential of red seaweed has been widely investigated. Sulphated polysaccharides, mainly carrageenans, present interesting antiviral properties, which are influenced by the processing conditions, the extraction stage, and eventual chemical modifications [170]. Kim et al. [118] extracted sulphated galectins conjugated with uronic acid from the red alga *Gyrodinium impudicum* to investigate their activity as anti-influenza agents. Results showed significant antiviral activity (IC_50_ 0.19–0.48 µg/mL), which is related to galectin’s ability to interact with viral particles, preventing virus adsorption and internalization.

Wang and co-workers [171] reported that the low molecular weight carrageenan oligosaccharide and their sulphated derivatives could effectively inhibit IAV, strain H1N1. Results showed that low molecular weight (LMW) carrageenans have a better antiviral action compared to high molecular weight (HMW) carrageenans. It has also been reported that carrageenan polysaccharides could enter the target cells and do not interfere with H1N1 adsorption, thus they inhibited influenza A virus infection by directly binding to the virus particles. LMW carrageenan oligosaccharides did not bind to the cell surface of infected cells but inhibited viral mRNA and protein expression after its internalization into cells. They affect virus replication after viral internalization, but prior to virus release in one replication cycle. Therefore, the authors suggested that the integration of carrageenan oligosaccharide in pharmaceuticals might be an alternative approach for anti-influenza A virus therapy [171].

In another study, the effect of low molecular weight ɩ-carrageenan oligosaccharides and their sulphated derivatives was investigated in IAV-infected mice. Results of both treatments evidenced a significantly improved survival rate and decrease in neuraminidase activity in the lungs, confirming the anti-influenza potential of carrageenan oligosaccharide in vivo [122]. The study revealed that k-carrageenan oligosaccharide was the most active with a molecular weight of 1–3 kDa.

Tang et al. [122] confirmed the effectiveness of low molecular weight carrageenans and their derivatives against influenza virus FM1-induced pulmonary edema in mice. Results of the in vivo experiment confirmed the best antiviral activity for 3 kDa k-carrageenan.

Yu et al. [121] suggested using HMW hybrid carrageenan (ɩ/κ/ν-carrageenan) from the red alga *Eucheuma denticulatum* as a potential inhibitor of IAV. Antiviral activity against H1N1 influenza virus was highest when the hybrid polysaccharide was used, and the H1N1 virus suppression index was 52% using a polysaccharide dose of the lowest molecular weight compared with other polysaccharides.

The idea of creating a new drug combining carrageenan and known antiviral drugs is an interesting one; Morokutti-Kurz et al. [123] proposed a combined intranasal spray including carrageenan and zanamivir (neuraminidase inhibitor) for the prevention and treatment of influenza. Their study showed that combinate therapies applied to mice before infection and 36 h after infection led to a rate of survival between 50–90%. Carrageenans were reported to develop a physical barrier in the nasal cavity against respiratory viruses, such as the influenza virus. This potential of carrageenan in nasal spray was found in the study of Koenighofer et al. [164]; patients with acute influenza were randomly provided with intranasal spray with or without ɩ-carrageenans. After two days, in patients treated with carrageenan, the disease regressed rapidly and the severity of symptoms was milder.

Leibbrand et al. [124] demonstrated the effectiveness of carrageenans against human influenza A viruses. The authors determined the sensitivity of k- and ɩ-carrageenan to H1N1 influenza virus strains, as well as the pandemic H3N2 strain, using the plaque formation method in canine kidney epithelial cells (MDCK). In this report we demonstrate that both k- and ɩ-carrageenan are potent inhibitors of influenza virus infectivity in vitro, protecting MDCK cells from virus-induced cell death. Carrageenans have also been tested in vivo on mice models. Survival of MDCK cells in the presence of ɩ-carrageenan up to 96 h post infection with H1N1 showed a dramatic reduction of viral titers, indicative of a protective effect of ɩ-carrageenan. Both subtypes of carrageenan showed antiviral activity, but ɩ-carrageenan showed higher antiviral activity at less concentration (IC_50_ = 0.04 µg/mL) compared with k-carrageenan (IC_50_ = 0.3 µg/mL).

Eccles et al. [166] published a study case in which patients with early symptoms of the common cold were subjected to placebo treatment and ɩ-carrageenan nasal spray for seven days. No serious adverse events were reported and there were no withdrawals due to adverse event development. Presented side effects were resolved, therefore no special actions were necessary. The small number of adverse event reports (vomiting, nausea and abdominal pain, loss of voice) supports the good safety-profile of ɩ-carrageenan. The authors showed a significant reduction in symptoms of the disease such as nasal congestion, runny nose, cough, and sneezing in patients subjected to carrageenan spray solution: nasal congestion at the end of the observation period was noted by 63.6% of persons in the placebo group and 28.6% of the group receiving carrageenan. A significant decrease was noted for the viral capacity in the nasal mucosa in patients treated with the spray (92%), while placebo treatment did not affect viral replication, therefore the authors considered ɩ-carrageenan nasal spray as a promising compound for safe and effective treatment of early symptoms of the common cold [166].

In a similar way, 211 patients suffering from early symptoms of the common cold were treated for seven days. A nasal spray with saline solution (for placebo group) and carrageenan (for treated group) was applied three times daily. Patients with cold virus infection detected the alleviation of symptoms 2.1 days faster in the carrageenan group in comparison to placebo, and viral titers in nasal fluids showed a significantly greater decrease in carrageenan patients between day 1 and day 3/4. The study demonstrated that carrageenan-based nasal spray reduced the expression of pro-inflammatory cytokines and increased the level of IL-1 and IL-12p40 receptor antagonists, which are known to have anti-inflammatory action in the nasal lavage of patients with respiratory viral infections. Therefore, a direct and local administration of carrageenan in adults with common cold virus at early symptoms can reduce the duration of cold symptoms [167].

The aim of the study of Shao et al. [125] was to investigate the antiviral activity of κ-carrageenan against the swine pandemic 2009 H1N1 influenza virus. MDCK cells were first infected with the SW731 strain, then treated with κ-carrageenan. It was observed that the cell viability was significantly promoted by κ-carrageenan in a dose-dependent way, confirming the anti-IAV activity of κ-carrageenan specifically for the inhibition of SW731 replication. The titer of influenza virus SW731 decreased in cases where the virus was treated with the polysaccharide before or during infection of the cells, suggesting that carrageenan acts at the extracellular level, binding to HA’s salic acid receptor, and intracellular stages of influenza virus replication.

A recent study of Jang et al. [126] examined the antiviral activity of λ-carrageenan loaded to influenza virus-infected MDCK cells. Carrageenan seems to target viral entry by directly attenuating the infectivity of the viral particles. The result from an in vitro bioassay suggested that λ-carrageenan could interact with a viral protein important for virus entry, possibly HA, suggesting that λ-carrageenan targets the attachment of influenza virus to its cell surface receptors by neutralizing viral glycoprotein HA. To investigate the antiviral activity of λ-carrageenan in vivo, mice were infected intranasally with polysaccharides. As a control, infected mice received oseltamivir phosphate orally twice a day for six days. Antiviral activity was determined by monitoring body weight and mortality for 15 days. The results revealed that intranasal administration of 5 mg/kg λ-carrageenan mitigated infection-mediated body weight loss, yielding a 60% survival rate, an effect not observed with 1 mg/kg. As expected, treatment with oseltamivir phosphate at 10 mg/kg/day for six days showed remarkable therapeutic effects. In conclusion, this data suggested that intranasal co-administration of λ-carrageenan and oseltamivir prevents viral infection-mediated body weight loss and reduces mortality [126].

Therefore, the antiviral activities of red algae polysaccharides are very broad, and they can suppress the replication of viruses with different mechanisms of actions which are associated with carrageenans, the virus serotypes and the host cell itself [70,99].

### 4.2. Anti-HIV Activity of Seaweeds

The sulphated fucans from the seaweed species *Dictyota mertensii*, *Lobophora variegata*, *Spatoglossum schroederi* and *Fucus vesiculosus* reported by Queiroz et al. [80] were able to inhibit the activity of HIV reverse transcriptase. Their study suggested that fucans antiviral activity is not only dependent on the sulphated groups, but also on the sugar rings that act to spatially orientate the charges in a configuration that recognizes the reverse transcriptase determining the specificity of the binding with the enzyme. Indeed, some studies suggested that other fucoidans characteristics could play a role in influencing their antiviral properties: the degree of polymerization, polymeric backbone, and carbohydrate portions, in fact the length of the sugar backbone and its structure can also act on reverse transcriptase activity [74,172]. Fucan from *S. schroederi* were desulphated and their antiviral activity measured; results showed that desulphated fucans exhibited low reverse transcriptase activity compared to sulphated fucans, supporting the hypothesis for which a higher number of sulphated groups increases the antiviral activity.

Fucoidan fractions were isolated from *Sargassum swartzii* to investigate in vitro anti-HIV-1 property. The fraction with greater sulphate content exhibited higher antiviral activity. This fucoidan fraction resulted in a >50% reduction in HIV-1 p24 antigen levels and reverse transcriptase activity, thus the pharmacodynamics of fucoidans consist in both inhibition of the virus by avoiding the center of the virus in host cells, and inhibition of the reverse transcriptase enzyme [71].

The investigation of Thuy et al. [128] explored the anti-HIV-1 activity of fucoidans from *S. mcclurei*, *S. polycystum* and *Turbinaria ornata*; they all displayed similar in vitro antiviral activities (average of IC_50_ ranging from 0.33 to 0.7 g/mL, no cytotoxicity revealed). This work showed that the antiviral activity is not given by the inhibition of reverse transcriptase, but fucoidans inhibited HIV-1 infection when they were pre-incubated with the virus, thus fucoidans blocked the early steps of HIV binding with target cells.

In the same way, galactofucans and fucans extracted from *Saccharina* sp. showed greater antiviral activity in suppression transduction of Jurkat cells by pseudo-HIV-1 particles, acting before the virus infect the host cell and have no effect on the reverse transcriptase [130]. Therefore, the molecular mechanism of action of seaweed’s compounds can vary depending on several factors, indeed, the antiviral properties exhibited by seaweed need further investigation and clinical trials.

Bioactive fucoidan fractions (CFF, FF1 and FF2) were isolated from *Sargassum swartzii* and their anti-HIV-1 property was investigated. Fraction FF2 was found to exhibit significant anti-HIV-1 activity at concentrations of 1.56 and 6.25 g/mL, as observed by >50% virus reduction, establishing the inhibitory effect of the polysaccharides on the p24 antigen and reverse transcriptase activity. Fucoidan fractions have no cytotoxic effects on human peripheral blood mononuclear cells (PBMC) at the concentration range of 1.56–1000 g/mL. The highest inhibitory activity (95.6 ± 1.1%) and inhibition of RT (78.9 ± 1.43%) was shown by the polysaccharide FF2 at a dose of 25 µg/mL. Through Fourier-Transform Infrared Spectroscopy (FT-IR) higher sulphate content in fraction FF2 has been detected, giving the authors the indication that higher anti-HIV activity is correlated with higher sulphation of fucoidan [71].

A series of galactofucan fractions obtained from the brown seaweed *Adenocystis utricularis* was analysed for in vitro anti-HIV-1 activity in human peripheral blood mononuclear infected cells. Results showed that two of the five fractions analysed had potent anti-HIV-1 activity on the replication of HIV-1 in low doses (IC_50_ = 0.6 and 0.9 µg/mL, respectively). From the test performed, no virucidal activity was detected, therefore the inhibitory effect was not due to an inactivating effect on the viral particle but by blocking the early stages of virus replication. From the results obtained, the authors recommend these substances as good candidates for the creation of prophylaxis and therapeutic treatments against HIV infection [131].

Sanniyasi et al. [129] examined the anti-HIV activity of fucoidans extracted from *D. bartayesiana* and *Turbinaria decurrens*. The authors found inhibition of HIV replication at an IC_50_ value of 1.56 µg/mL for *D. bartayesiana* and 3 µg/mL for *T. decurrens*, with inhibition of 92% and 89%, respectively, at maximum concentration with highly active HIV-inhibitory activity, confirming the effective retroviral inhibitor activity of sulphate polysaccharides.

A recent study published by Santo et al. [132] investigated the RT-HIV inhibition and antioxidant activities of crude extracts (methanolic, aqueous, and hot aqueous) from three Brazilian species: *S. vulgare* (Ochrophyta), *Palisada flagellifera* (Rhodophyta), and *Ulva fasciata* (Chlorophyta). All three seaweed extracts showed antioxidant activity, while only hot aqueous extracts from *S. vulgare* showed the highest anti-HIV potential. The recent study of Harb et al. [133] evaluated the potential of beach-cast seaweed methanolic and aqueous extracts to inhibit the reverse transcriptase enzyme of the HIV-1. In general, the aqueous extracts showed higher RT inhibition potential as an antiviral agent than methanolic extracts. However, both extracts from strand-beach algae *Alsidium seaforthii*, *Osmundaria obtusiloba*, *Dictyopteris jolyana*, and *Zonaria tournefortii* were highly promising, reaching inhibition above 90%. Furthermore, polyphenols and tannins have been reported as the main metabolites responsible for high antiviral activity in methanol extracts from red and brown algae, thus the combination of them or their singular action can explain the antiviral activity.

Another study involving the inhibition of HIV-1 reverse transcriptase has been recently published by Polo et al. [134]. The authors investigated the inhibitory activity against RT-HIV-1 of crude extracts from *S. filipendula* by using different concentrations of methanolic and aqueous extracts. Samples tested with aqueous extracts showed a higher antiviral activity, including samples treated with UV radiation. Even with the lowest extract concentration (50 μg/mL), all the extracts had close to 100% anti RT-HIV efficiency.

Polyphenols from *Ecklonia cava* have been reported to have an effect on the HIV virus. In particular, 6,6′-bieckol, a phlorotannin, showed high inhibition against HIV-1-induced syncytium formation, viral p24 antigen production and lytic effects, as well as the inhibition of HIV-1 reverse transcriptase in vitro [135]. The study of Ahn et al. [136] showed that 8,8-bieckol and 8,4-dieckol from *E. cava* inhibit in vitro reverse transcriptase and HIV-1 protease, while eckol and phlorofucofuroeckol A did not exhibit such activity.

Therefore, these positive results suggest polysaccharides and phlorotannins from brown algae as potential drug component candidates for development of a new generation of therapeutic agents against HIV, along with polysaccharides.

In 2008, a clinical study of carrageenan-based gel was allowed to establish its effectiveness as a means of blocking sexual HIV infection in women [173]. The clinical trial has been performed with 6202 sexually active, HIV-negative women aged 16 years and older. Patients were followed up for up to two years. They were randomly assigned with carrageenan gel treatment (*n* = 3103) or placebo. Results did not show carrageenan-based gel efficacy in prevention of male-to-female transmission of HIV, although no safety concerns were recorded. Results could be compromised by the poor adherence of a non-frequent use of the gel during sexual intercourse. In spite of this this negative outcome, the search for female-controlled HIV-prevention methods must continue in order to understand the factors that compromised the potential of carrageenan against the infection.

Carrageenan has been reported to have low anti-HIV activity as well in the study of Lynch et al. [174]; k-carrageenan binds T cells, avoiding the disruption of these cells by the HIV virus, but carrageenan was a relatively poor inhibitor of HIV infection, while a stronger activity was detected with a pentosan sulphate polysaccharide. Moreover, the strong anticoagulant activity of carrageenan is considered as an adverse reaction when used as a therapeutic drug for AIDS, therefore it is not the best compound for treating HIV diseases [97,175].

Griffithsin, a novel 121-amino-acid carbohydrate-binding protein from red algae *Griffithsia* sp., has been reported to have in vitro efficacy against HIV-1, preventing HIV entry into the host cells, cell fusion and cell-to-cell transmission of HIV [176]. The protein griffithsin has been further investigated for its potential antiviral activity against several viruses.

### 4.3. Anti-Herpetic Activity of Seaweeds

A significant number of results highlight the antiviral activity of seaweed compounds against Herpes viruses’ strains. Already at the end of the 20th century, Santos et al. [137] demonstrated the antiherpetic effect of aqueous cold extracts from brown algae *Padina gymnospora*, *Laminaria abyssalis*, and *Sargassum vulgare* against acyclovir resistant Herpes simplex virus-1, with 99%, 100%, and 92% inhibition respectively. Moreover, inhibitory activity against standard strains of HSV-1 and acyclovir resistant HSV-1 has been detected in the hot water extract of *Hydroclathrus clathratus* and *Lobophora variegata* [138].

Acyclovir is a synthetic purine nucleoside identified as the first antiviral drug to specifically target viral DNA polymerase and inhibit DNA chain elongation. It is one of the most effective and selective antiviral drugs, and it has an antiviral effect on HSV-1, HSV-2 and varicella zoster virus (VZV) by interfering with DNA synthesis and inhibiting viral replication [177]. It also is a safe and effective drug for vaginal administration, used in the treatment of primary or recurrent genital Herpes lesions. A clinical study by Corey et al. showed that topical acyclovir shortens the duration of viral shedding and accelerates the healing of some genital Herpes simplex virus infections, as well as preventing the transmission of genital Herpes [178].

A recent study from Sun et al. [149] shows that purified polysaccharides isolated from water extract of *S. henslowianum* exhibit anti-HSV-1 and anti-HSV-2 activity. The antiviral activity was enhanced when polysaccharides were added during virus infection, suggesting that the strong activity affects the early stages of virus infection, preventing the absorption from the virus to the host cell membrane. Cytotoxicity tests show low toxicity; therefore, the authors suggest the use of these polysaccharides as potential candidates for clinical applications in individual or combination drug therapy.

Fucoidans from brown algae have already showed a broad antiviral spectrum, and inhibition of HSV-1 has been confirmed pre-clinical test by Hayashi et al. [139] and Lee et al. [76], where both in vitro and in vivo tests showed anti-HSV-1 and anti-HSV-2 activity of fucoidan extracted from *Undaria pinnatifida*.

During a clinical study, two patients with Herpes labialis (commonly caused by HIV-1) were treated with Power Fucoidan Cream^TM^ (4% fucoidan cream), with fucoidans extract from Japanese algae *Nemacystus decipiens*. After topical administration for one week, the infection was significantly improved in terms of both time to healing and time to loss of discomfort. After further clinical trials, the product has been released in the market (https://goodsofjapan.com/, accessed on 4 April 2022) [168].

Among red algae, Soares et al. [144] investigated dichloromethane:methanol extracts from the Brazilian seaweeds *Stypopodium zonale*, *Corallina panizzoi*, *Jania crassa*, *Tricleocarpa cylindrica*, *Bostrychia radicans*, *Laurencia dendroidea*, *Osmundaria obtusiloba*, *Spyridia clavata*, *Pterocladia capillacea*, *Hypnea musciformis*, *Hypnea spinella*, *Chondracanthus acicularis*, and *Plocamium brasiliense* against HSV-1 and HSV-2 acyclovir-resistant strains. Results have shown an inhibition percentage ranging from 43.8 to 97.5%, with *Laurencia dendroidea* having the best inhibitory activity against HSV-1. In this study, also extract from green algae seaweed’s such as *Ulva fasciata, Codium decorticatum* and *Physocarpus capitatus* showed high activity against HSV-1. The green alga *Penicillus capitatus* and brown algae *Stypopodium zonale* were the most active against HSV-2 (96.0 and 95.8%). These genera had high concentrations of polysaccharides and fatty acids that might be responsible for the observed activity [144].

Anti-HSV-1 activity of sulphated galectins from the Rhodophyta *Pterocladia capillacea* was already investigated by Pujol et al. [145], and these compounds showed antiviral activity with an EC_50_ value ranging from 3.2 to 6.1 μg/mL^−1^. Anti-HSV-1 activity of hot water extract of *Hypnea musciformis* has also been confirmed in previous pre-clinical studies [146,150], where it expresses the highest inhibitory effect on HSV-1 with *Asparagopsis armata*, *Corallium rubrum*, *Gelidum spinulosum*, *Plocamium cartilagineum* and *Sphaerococcus coronopifolius*.

In a recent work from Pliego-Cortés et al. [147] the in vitro antiherpetic activity of sulphated polysaccharides extracted from seaweeds collected in France and Mexico from stranding events were evaluated. Results showed significant antiviral activity and no cytotoxicity exhibited from red alga *Halymenia floresii*. The strong biological activity was expressed when the polysaccharide and the virus were added simultaneously, thus it suggests that the polysaccharides block the entry of the virus into the cell.

Talarico et al. [148] in their study explore the in vitro antiviral activity against HSV-1 and HSV-2 of sulphated galactan crude extracts and the main fractions obtained from two red seaweeds collected in Brazil, *Gymnogongrus griffithsiae* and *Cryptonemia crenulata*. The results suggest that single k/ɩ/v-carrageenan and hybrids exhibited antiherpetic activity with IC_50_ at 50% in the range 0.5–5.6 µg/mL against HSV-1. A significant result was the protective effect of crude galactan preparation obtained from *C. crenulata* against HSV-2 vaginal infection in a murine model, suggesting the combined use of this low-cost product, which is easy to obtain in large quantities, for prophylaxis of virus infection treatments [148].

Study cases involving carrageenans extracted from *Gigartina skottsbergii* have been identified as potent inhibitors of HSV-1 by avoiding the internalization of the virus into the cell as Carlucci et al. [151] reported. The authors in further research [154] evaluated the protective effect of ʎ-carrageenan from *G. skottsbergii* in a murine model of Herpes simplex virus type 2 (HSV-2) genital infection. From the in vivo test, 100% protection against HSV-2 mortality and replication was achieved in a very strict model of murine infection at a high dose of virus, meaning it was a remarkable success. Moreover, neither virus nor neutralizing antibodies against HSV-2 were detected in serum until three weeks after infection, thus it is unlikely that protected surviving animals possess latent infection.

Over the last century, the potential of metal nanoparticles attracted researchers to explore their applications in biomedical sciences [179], as silver and gold nanoparticles have proved to have interesting interactions with biological targets, such as microbes and viruses [180,181,182,183]. On the other hand, *Sargassum* sp. are rich in polysaccharides that express interesting biological activities [184]. Therefore, Dhanasezhian et al. [152] investigated and evaluated the anti-HSV activity of gold (Au) and silver (Ag) nanoparticles synthesized by *S. withtii*. From cytotoxicity analysis, Au nanoparticles were found to be non-toxic to Vero cells at the four different concentrations tested, while Ag showed toxicity at higher concentrations but was non-toxic at two out of four concentrations tested. Both Au and Ag nanoparticles synthesized by *S. withtii* reduced cytopathic effects caused by HSV in a dose-dependent manner. Au nanoparticles reduced the cytopathic effects by 70% for HSV-1 and HSV-2 at 10 and 25 μL, whereas at concentrations that do not show cytotoxicity, Ag nanoparticles effectively reduced by 70% and 50% the cytopathic effects of HSV-1 and HSV-2 (1 μL), respectively, while at 2.5 μL Ag nanoparticles reduce the cytopathic effects for both HSV-1 and HSV-2 by 70%. Based on these observations, this study concludes that the preparation of metal nanoparticles integrated with seaweed’s polysaccharides could be a potential alternative for treating viral infections [152].

Vissani et al. [153] evaluated the antiviral activity in vitro against HSV-1 and HSV-2 and some Herpes viruses of veterinary interest such as equid Herpes virus 3(EHV3), Bovine Herpes virus 1 (BoHV1), Suid Herpes virus 1 (SuHV1) and Feline Herpes virus 1 (FeHV1). Antiviral tests have been performed on confluent monolayers of Ederm cells infected with these viral strains and simultaneously treated with λ-carrageenan extracted from *Gigartina skottsbergii*. From the results, the authors confirm the effectiveness in preventing infection by EHV3, BoHV-1, and SuHV1, as well as HSV-1 and HSV-2. The authors conclude that most likely λ-carrageenan binds to the envelope glycoprotein of the virus, preventing viral attachment to the cell surface receptor.

Polysaccharides extracted from *S. coronopifolius* and *Boergeseniella thuyoides* (Rhodophyta) collected on the coast of Morocco were tested against HSV-1. Results showed that the in vitro inhibition of HSV-1 replication on Vero cell values of EC_50_ of 4.1 and 17.2 µg/mL, respectively. Polysaccharides did not exert an important virucidal effect. Preincubation of the virus with the polysaccharide did not significantly protect Vero cells against HSV-1, while the EC_50_ obtained after two days of incubation increased. It might be possible that the sulphated polysaccharide could affect the virus-cell attachment by structural modification at the host cell membrane, which would alter virus-specific receptor sites. In this way, the virus will not enter the host cell. Since not too much information is available, further studies will be necessary to establish structure-activity relationships in antiviral activity [140].

ι-carrageenans from *Solieria chordalis* extracted with alkali extraction also showed higher antiviral activity comparable to that of acyclovir under the same conditions. Nevertheless, the antiviral activity and mechanism of action of *S. chordalis* against HSV-1 is not clear at present, and further studies are needed to clarify the relationship between chemical structure, properties, and anti-HSV-1 activity [141].

A recent study of Bedoux et al. [142] evaluated the in vitro antiherpetic activity of polysaccharides extracted from *Rhodymenia pseudopalmata*, *S. filiformis, Hydropuntia cornea* (Rhodophyta) and *Sargassum fluitans* (Phaeophyceae). Results showed that polysaccharides from *S. fluitans* (EC_50_ = 42.8 μg/mL) and *S. filiformis* (EC_50_ = 136.0 μg/mL) showed antiviral activity against HSV-1 on a Vero cells line. Biochemical analysis suggested that the enhanced antiviral activity might be due to the high grade of sulphation of these polysaccharides, while low sulphate content in the polysaccharide of *Hydropuntia cornea* could be related to the lack of antiviral activity.

The green seaweed *Codium fragile* and red seaweed *Chondrus crispus* were subject to enzymatic hydrolysis and the extracts obtained were tested for their antiviral activity on HSV-1 using Vero cell lines. *C. crispus* was characterized by higher levels of protein and sulfate, while *C. fragile* had a higher amount of neutral sugar and ash. The enzymatic extracts were tested for their antiviral activity and their cytotoxicity was also evaluated. After three days of treatment, no cytotoxicity was observed in extracts of either of the seaweed’s tested. Inhibition of viral activity was observed in the enzymatic extract of *C. fragile* (36.5 ± 10.3 μg/mL) and *C. crispus* (77.6 ± 9.6 μg/mL), while no anti-HSV-1 activity was observed in *C. crispus* hydrolysates obtained from enzymatic extraction. On the other end, extract of *C. fragile* subjected to enzymatic hydrolysis showed strong HSV-1 inhibition [143]. This could be explained as the percentage of glucose was significantly higher in the enzymatic extract of both seaweeds tested. Interestingly, derivatives of glucose have been reported as anti-HSV compounds.

In the investigation of Nixon et al. [155] the protein griffithsin isolated from the red algae *Griffithsia* sp. also displayed modest inhibitory activity against genital Herpes HSV-2 in mice treated with 0.1% griffithsin gel. Griffithsin, but not placebo gel, prevented viral spread, significantly reduced disease scores, and resulted in greater survival if present posteriorly to viral entry, and this was also demonstrated by Derby et al. [156] Levendosky et al. [157], and Tyo et al. [158]. These findings demonstrate that griffithsin inhibits not only HSV-2 but other viral strains such as HPV or HIV. Nevertheless, further studies and clinical trials need to be performed to assure the efficacy of griffithsin proteins.

### 4.4. Effect of Seaweeds Compounds on SARS-CoV-2

The emergent pandemic caused by SARS-CoV-2 in 2019 continues to spread around the world and remains a major public health threat. Lack of vaccines, or supplies, especially in undeveloped countries, intensify this issue, as does the fact that a non-negligible part of the population either refuse vaccination or cannot be vaccinated due to age, pre-existing illness or non-response to existing vaccines. Due to these factors, it is likely that new variants will emerge, and they will likely be more efficiently transmitted, more virulent and more capable of escaping naturally acquired and vaccine-induced immunity. The situation forced scientists and researchers to find treatments to reduce symptoms and the mortality rates of people affected by this new coronavirus and its variants [185].

Sulphated polysaccharides extracted from *Saccharina japonica* demonstrate in vitro inhibition of SARS-CoV-2. The polysaccharides extracted were RPI-27 and RPI-28, complex fucoidans, chemo-enzymatically synthesized tri-sulphated heparin, and unfractionated heparin. Among the tested polysaccharides, the most potent compound tested was RPI-27, which possesses high molecular weight and branched polysaccharides. Results suggested that sulphate polysaccharides bind tightly to the S protein of SARS-CoV-2, which suggests that they can act as decoys to interfere with S-protein binding to the heparan sulfate co-receptor in host tissues inhibiting viral infection [159].

In the study of Song et al. [160], the authors investigated the antiviral activity of fucoidan from brown algae and ɩ-carrageenan from red algae. These polysaccharides showed significant antiviral activity, inhibiting SARS-CoV-2 infection on Vero E6 cells at various concentrations between 3.90–500 μg/mL. It has been revealed that before SARS-CoV-2 entry into the host cell, spike protein binds to heparan sulphate chains. Therefore, S glycoprotein is the most possible target for the sulphated polysaccharides with structures like heparan sulphate, which is present in fucoidan. Therefore, it is likely that seaweed polysaccharides interacted with the S glycoprotein of SARS-CoV-2 to inhibit SARS-CoV-2 infection [160].

As previously mentioned, Jang et al. [126] evaluate the anti-influenza activity of λ-carrageenan. During the same study case, the authors evaluated as well if carrageenans can neutralize SARS-CoV-2. The antiviral assay revealed that λ-carrageenan suppresses the entry of SARS-CoV-2 in Vero cells infected in a dose-dependent manner. Carrageenans have intrinsic anti-coagulant activity, and thus any unwarranted side effects should be first analysed before clinical applications, as dysfunctional or aberrant coagulation is responsible for the hyper-inflammatory responses observed in severe cases of influenzas or SARS-CoV-2 infection-mediated pneumonia [186,187]; thus, with the presence of carrageenan, the anti-coagulant signals might result in over-stimulation, as it is already stimulated in the lungs of infected patients [126].

In the study of Fröba et al. [163], the authors investigated the antiviral effect of ɩ-, ʎ- and k-carrageenan extracted from red seaweed on SARS-CoV-2 Wuhan type and the spreading variants Alpha, Beta, Gamma, and Delta. Carrageenans were mixed with nasal and mouth sprays. The authors first determined whether ɩ-, ʎ- and k-carrageenans block the infection of cells from viral spike proteins from the SARS-CoV-2 and the variants Alpha, Beta, Gamma, and Delta with the same efficacy as for the Wuhan type. Therefore, A549ACE2/TMPRSS2 and Calu-3 human lung cells were infected with viral particles and spike driven infection was measured. The results showed that the highest antiviral activity was demonstrated by ɩ-carrageenan that inhibited the viral infection of SARS-CoV-2 Wuhan type in a similar way as the Alpha, Beta, Gamma, and Delta variants. k- and ʎ-carrageenan also showed some antiviral activity when used at 100 µg/mL but were hardly active at 10 µg/mL, while at the same concentration, ɩ-carrageenan almost completely blocked the production of progeny virions [163].

Another study case that involves anti-SARS-CoV-2 activity using ɩ-, k- and ʎ-carrageenan was conducted by Morokutti-Kurz et al. [161] in Vero B4 cells that were infected with coronavirus and were treated with carrageenans. As with the previous case, ɩ-carrageenan concentrations of 10 μg/mL or higher resulted in a reduction of the viral signal by more than 80%. Moreover, the presence of only 1 μg/mL ɩ-carrageenan resulted in a detectable reduction of infectivity by more than 20%. The authors also examined the high molecular weight fucoidan from *U. pinnatifida* and *F. vesiculosus*, which were effective in less than 50% reduction of infection at the higher concentration (100 μg/mL) [161].

The study of Varese et al. [162] assessed the in vitro effect of ɩ-carrageenan on the Calu-3 cell line, which comes from a submucosal adenocarcinoma of the bronchi, a line considered as a suitable in vitro model of the upper airway epithelium. The study revealed that sterile nasal spray solutions prepared with different concentrations of ɩ-carrageenan and sodium chloride inhibits infection of SARS-CoV-2 in Calu-3 cells in a dose-dependent manner, with an inhibition also present at low ɩ-carrageenan concentrations. Therefore, their data suggest that treatment with ɩ-carrageenan might also be positive in human patients affected by COVID-19, either in a prophylactic or therapeutic way.

A clinical trial was undertaken with a total of 400 clinically healthy physicians, nurses, kinesiologists, and other health care providers working in contact with patients hospitalized for COVID-19 [169]. Patients were treated with four daily doses of ɩ-carrageenan nasal spray or placebo for 21 days. The first outcome to be evaluated as a symptomatic illness was confirmed by detection of SARS-CoV-2 by reverse transcriptase–polymerase chain reaction (PCR). A Google Form survey with a structured questionnaire including the symptoms that should be reported was used, as patients had to monitor themselves daily. The investigator at the centre evaluated whether the symptoms could be assigned to a cause other than COVID- 19; under suspicious symptoms of COVID-19, the participants were sent to undergo a nasopharyngeal swab with a PCR test for SARS-CoV-2 and were isolated on a preventive basis until the PCR result was available. Patients with a positive PCR continued in isolation for management of their disease. PCR-negative individuals returned to their workplace 48 h after their symptoms had disappeared, while participants with a negative PCR whose symptoms persisted for 48 h after the first PCR had to undergo a new PCR. As he results showed, six participants were excluded from the final analysis because they had symptoms suggestive of COVID-19 at the time of randomization. Of the remaining 394 participants, 196 had been assigned to receive ɩ-carrageenan and 198 placebos. Thirteen individuals in the ɩ-carrageenan group and 14 in the placebo group withdrew their consent before the last day. From all the remaining participants, forty-three participants underwent a PCR test due to the presence of symptoms suggestive of COVID-19 (Table 2), and 31 were negative (7.6% in the ɩ-carrageenan group and 8.6% in the placebo group). Overall, new COVID-19 (symptomatic with confirmed PCR) developed in 12 of 394 participants (3.04%) during the 21 days of follow-up. The incidence of COVID-19 differs significantly between subjects receiving the nasal spray with ɩ-carrageenan (two of 196, 1.0%) and those receiving placebo (10 of 198, 5.0%), suggesting the use of carrageenans as good prophylactic treatment for preventing SARS-CoV-2 infection [169].

Moreover, among symptoms of SARS-CoV-2, gastrointestinal disorder has been reported by 61% of patients [188]. In such circumstances, algae-based bioactive metabolites could be used as food supplements capable of improving the gut microbiota, and thus potentially reducing the incidence of SARS-CoV-ACE2-associated gut microbiota symbiosis playing a key role in improving antiviral immunity by stimulating interferon production, decreasing immunopathology, and increasing natural killer (NK) cytotoxicity in COVID-19 patients [189]. Seaweed is rich in vitamins and minerals that can be used as dietary supplements for COVID-19 patients; the integration of marine bioactive metabolites in antiviral treatments can enhance the human gut microbiota integrity and maintain host health by controlling proper metabolism, epithelial barrier integrity, and immune system efficacy when used as prebiotics and nutritional food supplements. Furthermore, seaweed’s compounds already showed promising immunomodulation activity [190]. Therefore, further investigations are needed to be performed, with the scope to examine the pharmacokinetics and clarify the antiviral mechanisms once the structure of the marine polysaccharides is analysed. Considering the actual scenario and the potential of algal metabolites, the development of new antiviral formulations to cure viral infections is a real possibility [185]. It is necessary to implement the evaluation and research of algal bio-compounds. Only through the screening of a high number of bioactive compounds might the development of new natural drugs be possible.

## 5. Conclusions

The present review highlighted pre-clinical studies that involved seaweed’s bioactive compounds with the aim of investigating their antiviral activity. As several studies undertaken have reported, seaweeds are potential sources for natural antiviral molecules that might be integrated into new natural antiviral treatments, even though they possess pros and cons that need to be considered (Table 3). Each compound acts in a different way depending on its characteristics and those of the target virus, therefore future investigations with regard to seaweeds need to be undertaken to better know the potentiality of their bioactive compounds. It is clear from the pre-clinical and clinical tests performed that fucoidans possess a broad antiviral spectrum, making brown algae interesting candidates for pharmaceutical applications. Red algae also possess interesting antiviral properties, especially for *Herpes* viruses, and they could be used in combination with antiherpetic drugs as well to prevent sexually transmitted diseases, in addition to the incorporation of the griffithsin protein.

Research focused on the antiviral properties of seaweed’s active compounds is happening rapidly, as investigations carried out until now gave optimal results and described seaweed’s compounds as potential antiviral coadjutants. Clinical trials have been carried out to test the antiviral efficacy of seaweed’s bioactive compounds [164,166,167,169,191]. Indeed, carrageenan-based treatments have been approved for marketing in the EU, Asia and Australia as part of prophylaxis products to treat the common cold and related diseases [192], but they have not been approved in Europe.

The use of natural compounds as a replacement for synthetic ones or in combination with pre-existing antiviral treatments could be a way to optimize the production cost of drugs, to reduce side effects in patients as seaweed’s compounds present low-cytotoxicity, and to shift towards more natural, sustainable, inexpensive pharmaceutical products that could also be accessible in underdeveloped countries, where populations are often affected by viral epidemics, and they cannot access the necessary treatments to ensure a better quality of life due to their challenging economic situations.

## Figures and Tables

**Table 1:** Pre-clinical studies on antiviral activity of seaweed’s bioactive compounds against influenza virus, *Lentivirus*, HSVs, SARS-CoV-2.

Virus Investigated	Source of Antiviral Compound	Study Case	Compound	Reference
IAV	*Undaria pinnatifida*	Pre-clinical test: in vitro/in vivo	Fucoidan	[115]
	*Kjellmaniella crassifolia*	Pre-clinical test: in vitro	Fucoidan	[116]
	*Undaria pinnatifida*	Pre-clinical test: in vivo	Fucoidan	[117]
	*Gyrodinium impudicum*	Pre-clinical test: in vitro	sulphated galactan	[118]
	*Ecklonia cava*	Pre-clinical test: in vitro	Eckol, 7-phloreckol, phlorofucofuroeckol A, dieckol	[119]
	*Ecklonia cava*	Pre-clinical test: in vitro	phlorofucofuroeckol A	[120]
	*Eucheuma denticulatum*	Pre-clinical test: in vitro	ɩ/κ/ν-carrageenan	[121]
	Purchased carrageenan	Pre-clinical test: in vivo	k-carrageenan	[122]
	Purchased carrageenan	Pre-clinical test: in vivo	Carrageenan + zanamivir	[123]
	Purchased carrageenan	Pre-clinical test: in vitro/in vivo	k/ɩ-carrageenan	[124]
	Purchased carrageenan	Pre-clinical test: in vitro	κ-carrageenan	[125]
	Purchased carrageenan	Pre-clinical test: in vitro/in vivo	λ-carrageenan	[126]
Avian influenza viruses (H5N3, H7N2)	*Undaria pinnafitida*	Pre-clinical test: in vivo	Fucogalactan	[127]
HIV-1	*Dictyota mertensii, Lobophora variegata, Spatoglossum schroederi, Fucus vesiculosus*	Pre-clinical test: in vitro	Sulphated fucan	[80]
	*Sargassum swartzii*	Pre-clinical test: in vitro	Fucoidan	[71]
	*Sargassum mcclurei, Sargassum polycystum and Turbinaria ornata*	Pre-clinical test: in vitro	Fucoidan	[128]
	*Sargassum swartzii*	Pre-clinical test: in vitro	Fucoidan	[71]
	*Dictyota bartayesiana, Turbinaria decurrens*	Pre-clinical test: in vitro	Fucoidan	[129]
	*Saccharina* sp.	Pre-clinical test: in vitro	Galactofucan, fucan	[130]
	*Adenocystis utricularis*	Pre-clinical test: in vitro	Galactofucan	[131]
	*Sargassum vulgare*	Pre-clinical test: in vitro	Crude extracts	[132]
	*Alsidium seaforthii, Osmundaria obtusiloba, Dictyopteris jolyana, Zonaria tournefortii*	Pre-clinical test: in vitro	Crude extracts	[133]
	*Sargassum filipendula*	Pre-clinical test: in vitro	Crude extracts	[134]
	*Ecklonia cava*	Pre-clinical test: in vitro	6,6′-bieckol	[135]
	*Ecklonia cava*	Pre-clinical test: in vitro	8,8-bieckol, 8,4-dieckol	[136]
HSV-1	*Padina gymnospora, Laminaria abyssalis, Sargassum vulgare*	Pre-clinical test: in vitro	Crude water extract	[137]
	*Hydroclathrus clathratus, Lobophora variegata*	Pre-clinical test: in vitro	Crude water extract	[138]
	*Undaria pinnatifida*	Pre-clinical test: in vitro/in vivo	Fucoidan	[139]
	*Sphaerococcus coronopifolius, Boergeseniella thuyoides*	Pre-clinical test: in vitro	Sulphated polysaccharide	[140]
	*Solieria chordalis*	Pre-clinical test: in vitro	ι-carrageenan	[141]
	*Solieria filiformis, Sargassum fluitans*	Pre-clinical test: in vitro	Sulphated polysaccharide	[142]
	*Codimum fragile, Chondrus crispus*	Pre-clinical test: in vitro	Enzymatic extract	[143]
	*Stypopodium zonale, Corallina panizzoi, Jania crassa, Tricleocarpa cylindrica, Bostrychia radicans, Laurencia dendroidea, Osmundaria obtusiloba, Spyridia clavata, Pterocladia capillacea, Hypnea musciformis, Hypnea spinella, Chondracanthus acicularis, Plocamium brasiliense*	Pre-clinical test: in vitro	Dichloromethane extracts	[144]
	*Pterocladia capillacea*	Pre-clinical test: in vitro	Sulphated galactans	[145]
	*Hypnea musciformis, Asparagopsis armata, Corallium rubrum, Gelidum spinulosum, Plocamium cartilagineum, Sphaerococcus coronopifolius coronopifolius*	Pre-clinical test: in vitro	Crude extract	[146]
	*Halymenia floresii*	Pre-clinical test: in vitro	Sulphated polysaccharides	[147]
	*Gymnogongrus griffithsiae*	Pre-clinical test: in vitro	k/ɩ/v-carrageenan	[148]
HSV-1, HSV-2	*Sargassum henslowianum*	Pre-clinical test: in vitro	Fucoidan	[149]
	*Undaria pinnatifida*	Pre-clinical test: in vitro	Fucoidan	[76]
	*Penicillus capitatus, Stypopodium zonale*	Pre-clinical test: in vitro	Dichloromethane:methanol extract	[144]
	*Hypnea musciformis*	Pre-clinical test: in vitro	Crude extract	[150]
	*Cryptonemia crenulata*	Pre-clinical test: in vitro/vivo	Crude galactans	[148]
	*Gigartina skottsbergii*	Pre-clinical test: in vitro	Carrageenan	[151]
	*Sargassum withtii*	Pre-clinical test: in vitro	Au/Ag-nanoparticles synthesized by seaweed extract	[152]
HSV-1, HSV-2, EHV3, BoHV1, SuHV1, FeHV1	*Gigartina skottsbergii*	Pre-clinical test: in vitro	λ-carrageenan	[153]
HSV-2	*Gigartina skottsbergii*	Pre-clinical test: in vivo	ʎ-carrageenan	[154]
	*Griffithsia* sp.	Pre-clinical test: in vitro/vivo	Griffithsin	[155,156,157,158]
SARS-CoV-2	*Saccharina japonica*	Pre-clinical test: in vitro	Fucoidans (RPI-27, RPI-28)	[159]
	Purchased fucoidan	Pre-clinical test: in vitro	Fucoidan	[160]
	Purchased carrageenan	Pre-clinical test: in vitro	ɩ-carrageenan	
	Purchased carrageenan	Pre-clinical test: in vitro	λ-carrageenan	[126]
	Purchased carrageenan	Pre-clinical test: in vitro	k/ɩ/ʎ-carrageenan	[161]
	*Undaria pinnatifida, Fucus vesiculosus*	Pre-clinical test: in vitro	Fucoidan	[161]
	Purchased carrageenan	Pre-clinical test: in vitro	ɩ-carrageenan	[162]
SARS-CoV-2 and variants (Alpha, Beta, Gamma, Delta)	Purchased carrageenan	Pre-clinical test: in vitro	k/ɩ/ʎ-carrageenan	[163]

**Table 2:** Clinical studies on antiviral activity of seaweed’s bioactive compounds against influenza virus, HSVs, SARS-CoV-2.

Virus Investigated	Bioactive Compound	Clinical Study	Actions	Reference
IAV	ɩ-carrageenan	Clinical test: 254 patients	Regress of and severe symptoms of influenza	[164]
	Fucoidan	Clinical test: 70 patients	Higher antibody titers after fucoidan uptake	[165]
	ɩ-carrageenan	Clinical test: 35 patients	Decrease for the viral capacity in the nasal mucosa in patients treated with carrageenan-spray, while placebo treatment did not affect viral replication	[166]
	Carrageenan	Clinical test: 211 patients	Carrageenan-based nasal spray reduced the expression of pro-inflammatory cytokines and increased the level of IL-1 and IL-12p40 receptor antagonists (anti-inflammatory action)	[167]
HSV-1	Fucoidan	Clinical test: 2 patients	Infection, healing process and physical discomfort improved after 1 week of fucoidan-cream administration	[168]
SARS-CoV-2	ɩ-carrageenan	Clinical test: 400 patients	Decrease in COVID-19 Incidence of COVID-19 differs significantly between subjects receiving the nasal spray with ɩ-carrageenan	[169]

**Table 3:** Clinical studies on antiviral activity of seaweed’s bioactive compounds against influenza virus, HSVs, SARS-CoV-2.

Seaweeds Compounds as Antivirals against Enveloped Viruses		Reference
Pros	Broad antiviral spectrum	[113,156,157,158]
	In vitro/in vivo inhibition of virus replication	[115,122,124]
	Enhanced antibody production and immunomodulation activity	[127,165,189,190]
	Anti-inflammatory activity	[167]
	Prevention of the virus entry into the host cell	[126,131,149]
	RT inhibition potential	[132,133,134,136]
	No cytotoxicity effect	[128,143,147,149]
	Compounds can be included in prophylaxis treatments	[131,148,169]
Cons	Antiviral activity depends on the chemical structure (e.g., low antiviral activity is given by low number of sulphated groups)	[70,80,98,99,100,142]
	Strong anticoagulant activity of carrageenan could provoke adverse reaction in antiviral treatments	[97,126,175,186,187]
	Antiviral activity is influenced by extraction methods	[133,134]
	Poor adherence/non-frequent use of seaweed-based treatment decreases antiviral activity	[173]

## Data Availability

Not applicable.

## References

[1] Jabłonowska E., Pulik P., Kalinowska A., Gąsiorowski J., Parczewski M., Bociąga-Jasik M., Pulik Ł., Siwak E., Wójcik K. (2017). Efficacy and safety of nucleoside-sparing regimen based on raltegravir and ritonavir-boosted darunavir in HIV-1-infected treatment-experienced patients. J. Med. Virol..

[2] Marchetti M., Pisani S., Pietropaolo V., Seganti L., Nicoletti R., Orsi N. (1995). Inhibition of Herpes Simplex Virus Infection by Negatively Charged and Neutral Carbohydrate Polymers. J. Chemother..

[3] Firquet S., Beaujard S., Lobert P.E., Sané F., Caloone D., Izard D., Hober D. (2015). Survival of enveloped and non-enveloped viruses on inanimate surfaces. Microbes Environ..

[4] Abdullah A.A., Abdullah R., Nazariah Z.A., Balakrishnan K.N., Abdullah F.F.J., Bala J.A., Mohd-Lila M.A. (2018). Cyclophilin a as a target in the treatment of cytomegalovirus infections. Antivir. Chem. Chemother..

[5] Andersen P.I., Ianevski A., Lysvand H., Vitkauskiene A., Oksenych V., Bjørås M., Telling K., Lutsar I., Dumpis U., Irie Y. (2020). Discovery and development of safe-in-man broad-spectrum antiviral agents. Int. J. Infect. Dis..

[6] Mishra S., Pandey A., Manvati S. (2020). Coumarin: An emerging antiviral agent. Heliyon.

[7] Ianevski A., Zusinaite E., Kuivanen S., Strand M., Lysvand H., Teppor M., Kakkola L., Paavilainen H., Laajala M., Kallio-Kokko H. (2018). Novel activities of safe-in-human broad-spectrum antiviral agents. Antivir. Res..

[8] Gutierrez-Chamorro L., Felip E., Ezeonwumelu I.J., Margelí M., Ballana E. (2021). Cyclin-dependent Kinases as Emerging Targets for Developing Novel Antiviral Therapeutics. Trends Microbiol..

[9] Yoo S.J., Kwon T., Lyoo Y.S. (2018). Challenges of influenza A viruses in humans and animals and current animal vaccines as an effective control measure. Clin. Exp. Vaccine Res..

[10] Yu X., Wang C., Chen T., Zhang W., Yu H., Shu Y., Hu W., Wang X. (2017). Excess pneumonia and influenza mortality attributable to seasonal influenza in subtropical Shanghai, China. Infect. Dis..

[11] Koutsakos M., Nguyen T.H.O., Barclay W.S., Kedzierska K. (2015). Knowns and unknowns of influenza B viruses. Future Microbiol..

[12] Chen G., Shih S., Hsiao M., Chang S., Lin S., Sun C., Tsao K. (2007). Multiple Genotypes of Influenza B Viruses Cocirculated in Taiwan in 2004 and 2005. J. Clin. Microbiol..

[13] Shim J.M., Kim J., Tenson T., Min J.Y., Kainov D.E. (2017). Influenza virus infection, interferon response, viral counter-response and apoptosis. Viruses.

[14] Njouom R., Monamele G.C., Ermetal B., Tchatchouang S., Moyo-tetang S., Mccauley J.W., Daniels R.S. (2019). Detection of Influenza C Virus Infection among Hospitalized Patients, Cameroon. Emerg. Infect. Dis..

[15] Foni E., Chiapponi C., Baioni L., Zanni I., Merenda M., Rosignoli C., Kyriakis C.S., Luini M.V., Mandola M.L., Nigrelli A.D. (2017). Influenza D in Italy: Towards a better understanding of an emerging viral infection in swine. Sci. Rep..

[16] Werner J.L., Steele C., Werner J.L., Steele C. (2022). Innate Receptors and Cellular Defense against Pulmonary Infections. J. Immunol..

[17] Ezzat D., Aziz A., Amany M., Elawamry A.E.A.I. (2012). Comparison of Ocular Findings in Patients with H1N1 Influenza Infection versus Patients Receiving Influenza Vaccine during a Pandemic. Ophthalmic Res..

[18] White J.M., Whittaker G.R. (2016). Fusion of Enveloped Viruses in Endosomes. Traffic.

[19] Banerjee I., Miyake Y., Philip S.N., Schneider C., Horvath P., Kopf M., Patrick M., Ari H., Yamauchi Y. (2014). During Influenza A virus uses the aggresome processing machinery for host cell entry. Science.

[20] Pumroy R.A., Ke S., Zachariae U., Cingolani G., Pumroy R.A., Ke S., Hart D.J., Zachariae U., Cingolani G. (2015). Molecular Determinants for Nuclear Import of Influenza A PB2 by Importin a Isoforms 3 and 7 Article Molecular Determinants for Nuclear Import of Influenza A PB2 by Importin a Isoforms 3 and 7. Structure.

[21] Reguera J., Gerlach P., Cusack S. (2016). Towards a structural understanding of RNA synthesis by negative strand RNA viral polymerases. Curr. Opin. Struct. Biol..

[22] Lai A.L., Park H., White J.M., Tamm L.K. (2006). Fusion Peptide of Influenza Hemagglutinin Requires a Fixed Angle Boomerang Structure for Activity. J. Biol. Chem..

[23] Lousa D., Soares C.M. (2021). Molecular mechanisms of the influenza fusion peptide: Insights from experimental and simulation studies. FEBS Open Bio..

[24] Belanov S.S., Bychkov D., Benner C., Ripatti S., Ojala T., Kankainen M., Lee H.K., Tang J.W., Kainov D.E. (2015). Genome-Wide Analysis of Evolutionary Markers of Human Influenza A(H1N1)pdm09 and A(H3N2) Viruses May Guide Selection of Vaccine Strain Candidates. Genome Biol. Evol..

[25] Bouvier N.M., Palese P. (2008). The biology of influenza viruses. Vaccine.

[26] Bai Y., Jones J.C., Wong S.S., Zanin M. (2021). Antivirals targeting the surface glycoproteins of influenza virus: Mechanisms of action and resistance. Viruses.

[27] Vo T.S., Kim S.K. (2010). Potential anti-HIV agents from marine resources: An overview. Mar. Drugs.

[28] Fanales-Belasio E., Raimondo M., Suligoi B., Buttò S. (2010). HIV virology and pathogenetic mechanisms of infection: A brief overview. Ann. Ist. Super. Sanita.

[29] Lucas S.B., Hounnoun A., Peacock C., Beaumel A., Djomand G., N’Gbichi J.-M., Yeboeu K., Hondé M., Diomande M., Giordano C. (1993). The mortality and pathology of HIV infection in a West African city. AIDS.

[30] Vijayan K.V., Karthigeyan K.P., Tripathi S.P., Hanna L.E., de Mendoza C., Lozano A.B., Caballero E., Cabezas T., Ramos J.M., Soriano V. (2017). Pathophysiology of CD^4+^ T-Cell depletion in HIV-1 and HIV-2 infections. Front. Immunol..

[31] De Mendoza C., Lozano A.B., Caballero E., Cabezas T., Ramos J.M., Soriano V. (2020). Antiretroviral therapy for HIV-2 infection in non-endemic regions. AIDS Rev..

[32] Hilton W., Joanne M., Jim T., Tumani C., Sehu S., Joe B., Ngom P.T., Rolfe M., Wilkins A. (1994). THIV-2 infected patients surviver longer than HIV-1 infected patients. AIDS.

[33] Wilen C.B., Tilton J.C., Doms R.W. (2012). HIV: Cell Binding and Entry. Cold Spring Harb. Perspect. Med..

[34] Rein A. (2019). RNA Packaging in HIV. Trends Microbiol..

[35] Pornillos O., Ganser-pornillos B.K., Yeager M. (2011). Atomic-level modelling of the HIV capsid. Nature.

[36] Kleinpeter A.B., Freed E.O. (2020). HIV-1 maturation: Lessons learned from inhibitors. Viruses.

[37] Kimberlin D.W., Rouse D.J. (2004). Genital herpes. N. Engl. J. Med..

[38] Wu Y., Yang Q., Wang M., Chen S., Jia R., Yang Q., Zhu D., Liu M., Zhao X., Zhang S. (2021). Multifaceted Roles of ICP22/ORF63 Proteins in the Life Cycle of Human Herpesviruses. Front. Microbiol..

[39] Knipe D.M., Cliffe A. (2008). Chromatin control of herpes simplex virus lytic and latent infection. Nat. Rev. Microbiol..

[40] Pires de Mello C.P., Bloom D.C., Paixão I.C. (2016). Herpes simplex virus type-1: Replication, latency, reactivation and its antiviral targets. Antivir Ther..

[41] Riaz A. (2017). Recent Understanding of the Classification and Life Cycle of Herpesviruses—A Review. Sci. Lett..

[42] Münz C. (2019). Latency and lytic replication in Epstein–Barr virus-associated oncogenesis. Nat. Rev. Microbiol..

[43] Cohen J.I. (2020). Herpesvirus latency. J. Clin. Investig..

[44] Glaunsinger B.A. (2015). Modulation of the Translational Landscape during Herpesvirus Infection. Annu. Rev. Virol..

[45] Lv Y., Zhou S., Gao S., Deng H. (2019). Remodeling of host membranes during herpesvirus assembly and egress. Protein Cell.

[46] Heldwein E.E., Krummenacher C. (2008). Review Entry of herpesviruses into mammalian cells. Cell. Mol. Life Sci..

[47] Gianni T., Salvioli S., Chesnokova L.S., Hutt-fletcher L.M., Campadelli-fiume G. (2013). αvβ6-and αvβ8-integrins serve as interchangeable receptors for HSV gH/gL to promote endocytosis and activation of membrane fusion. PLoS Pathog..

[48] Aggarwal A., Miranda-Saksena M., Boadle R.A., Kelly B.J., Diefenbach R.J., Alam W., Cunningham A.L. (2012). Ultrastructural Visualization of Individual Tegument Protein Dissociation during Entry of Herpes Simplex Virus 1 into Human and Rat Dorsal Root Ganglion Neurons. J. Virol..

[49] Liu T., Du T., Evilevitch A., Brandariz-Nuñez A. (2019). Pressure-driven release of viral genome into a host nucleus is a mechanism leading to herpes infection. eLife.

[50] Wysocka J., Herr W. (2003). The herpes simplex virus VP16-induced complex: The makings of a regulatory switch. Trends Biochem. Sci..

[51] Turcotte S., Lippe R. (2005). Herpes Simplex Virus Type 1 Capsids Transit by the Trans-Golgi Network, Where Viral Glycoproteins Accumulate Independently of Capsid Egress. J. Virol..

[52] Hollinshead M., Johns H.L., Sayers C.L., Gonzalez-lopez C., Smith G.L., Elliott G. (2012). Endocytic tubules regulated by Rab GTPases 5 and 11 are used for envelopment of herpes simplex virus. EMBO J..

[53] Bialy D., Buch A., Mu O., Ivanova L., Do K., Bosse J.B., Pohlmann A., Binz A., Hegemann M., Nagel H. (2017). Inner tegument proteins of Herpes Simplex Virus are sufficient for intracellular capsid motility in neurons but not for axonal targeting. PLoS Pathog..

[54] Zhu S., Viejo-Borbolla A. (2021). Pathogenesis and virulence of herpes simplex virus. Virulence.

[55] Aneja K.K., Yuan Y. (2017). Reactivation and lytic replication of Kaposi’s sarcoma-associated herpesvirus: An update. Front. Microbiol..

[56] Bloom D.C. (2016). Alphaherpesvirus Latency: A Dynamic State of Transcription and Reactivation.

[57] Broussard G., Damania B. (2020). Regulation of KSHV Latency and Lytic Reactivation. Viruses.

[58] Cohen E.M., Avital N., Shamay M., Kobiler O. (2020). Abortive herpes simplex virus infection of nonneuronal cells results in quiescent viral genomes that can reactivate. Proc. Natl. Acad. Sci. USA.

[59] Hafezi W., Lorentzen E.U., Eing B.R., Muller M., King N.J.C., Klupp B., Mettenleiter T.C., Kuhn J. (2012). Entry of Herpes Simplex Virus Type 1 (HSV-1) into the Distal Axons of Trigeminal Neurons Favors the Onset of Nonproductive, Silent Infection. PLoS Pathog..

[60] Ren S., Koyuncu O.O., Greco T.M., Diner B.A., Cristea I.M., Enquist L.W. (2016). Two Modes of the Axonal Interferon Response Limit Alphaherpesvirus Neuroinvasion. Am. Soc. Microbiol..

[61] Koyuncu O.O., Song R., Greco T.M., Cristea I.M., Enquist L.W. (2015). The Number of Alphaherpesvirus Particles Infecting Axons and the Axonal Protein Repertoire Determines the Outcome of Neuronal. ASM J..

[62] Giovanetti M., Benedetti F., Campisi G., Ciccozzi A., Fabris S., Ceccarelli G., Tambone V., Caruso A., Angeletti S., Zella D. (2021). Evolution patterns of SARS-CoV-2: Snapshot on its genome variants. Biochem. Biophys. Res. Commun..

[63] V’kovski P., Kratzel A., Steiner S., Stalder H., Thiel V. (2021). Coronavirus biology and replication: Implications for SARS-CoV-2. Nat. Rev. Microbiol..

[64] Wang M.Y., Zhao R., Gao L.J., Gao X.F., Wang D.P., Cao J.M. (2020). SARS-CoV-2: Structure, Biology and Structure-Based Therapeutics Development. Front. Cell. Infect. Microbiol..

[65] Ignatius T.S., Yu M.B., Yuguo L., Tze W.W., Chan A.T., Lee J.H.W., Leung D.Y.C., Ho T. (2004). Evidence of Airborne Transmission of the Severe Acute Respiratory Syndrome Virus. N. Engl. J. Med..

[66] Otter J.A., Donskey C., Yezli S., Douthwaite S., Goldenberg S.D., Weber D.J. (2016). Transmission of SARS and MERS coronaviruses and influenza virus in healthcare settings: The possible role of dry surface contamination. J. Hosp. Infect..

[67] Li Y., Huang X., Yu I.T.S., Wong T.W., Qian H. (2004). Role of air distribution in SARS transmission during the largest nosocomial outbreak in Hong Kong. Indoor Air.

[68] Callahan L.N., Phelan M., Mallinson M., Norcross M.A. (1991). Dextran sulfate blocks antibody binding to the principal neutralizing domain of human immunodeficiency virus type 1 without interfering with gp120-CD4 interactions. J. Virol..

[69] Mandal P., Mateu C.G., Chattopadhyay K., Pujol C.A., Damonte E.B., Ray B. (2007). Structural features and antiviral activity of sulphated fucans from the brown seaweed *Cystoseira indica*. Antivir. Chem. Chemother..

[70] Damonte E., Matulewicz M., Cerezo A. (2004). Sulfated Seaweed Polysaccharides as Antiviral Agents. Curr. Med. Chem..

[71] Dinesh S., Menon T., Hanna L.E., Suresh V., Sathuvan M., Manikannan M. (2016). In vitro anti-HIV-1 activity of fucoidan from *Sargassum swartzii*. Int. J. Biol. Macromol..

[72] Iqbal M., Flick-Smith H., McCauley J.W. (2000). Interactions of bovine viral diarrhoea virus glycoprotein E(rns) with cell surface glycosaminoglycans. J. Gen. Virol..

[73] Jiao G., Yu G., Wang W., Zhao X., Zhang J., Ewart S.H. (2012). Properties of polysaccharides in several seaweeds from Atlantic Canada and their potential anti-influenza viral activities. J. Ocean Univ. China.

[74] Witvrouw M., De Clercq E. (1997). Sulfated polysaccharides extracted from sea algae as potential antiviral drugs. Gen. Pharmacol..

[75] Zhao Y., Zheng Y., Wang J., Ma S., Yu Y., White W.L., Yang S., Yang F., Lu J. (2018). Fucoidan extracted from *Undaria pinnatifida*: Source for nutraceuticals/functional foods. Mar. Drugs.

[76] Lee J.B., Hayashi K., Hashimoto M., Nakano T., Hayashi T. (2004). Novel antiviral fucoidan from sporophyll of *Undaria pinnatifida* (Mekabu). Chem. Pharm. Bull..

[77] Harden E.A., Falshaw R., Carnachan S.M., Kern E.R., Prichard M.N. (2009). Virucidal activity of polysaccharide extracts from four algal species against herpes simplex virus. Antivir. Res..

[78] Sun T., Zhang X., Miao Y., Zhou Y., Shi J., Yan M., Chen A. (2018). Studies on Antiviral and Immuno-Regulation Activity of Low Molecular Weight Fucoidan from *Laminaria japonica*. J. Ocean Univ. China.

[79] Baqai F.P., Gridley D.S., Slater J.M., Luo-Owen X., Stodieck L.S., Ferguson V., Chapes S.K., Pecaut M.J. (2009). Effects of spaceflight on innate immune function and antioxidant gene expression. J. Appl. Physiol..

[80] Queiroz K.C.S., Medeiros V.P., Queiroz L.S., Abreu L.R.D., Rocha H.A.O., Ferreira C.V., Jucá M.B., Aoyama H., Leite E.L. (2008). Inhibition of reverse transcriptase activity of HIV by polysaccharides of brown algae. Biomed. Pharmacother..

[81] Ahmadi A., Zorofchian Moghadamtousi S., Abubakar S., Zandi K. (2015). Antiviral Potential of Algae Polysaccharides Isolated from Marine Sources: A Review. BioMed Res. Int..

[82] Muto S., Niimura K., Oohara M., Oguchi Y., Matsunaga K., Hirose K., Kakuchi J., Sugita N., Furusho T., Et A. (1988). Polysaccharides from Marine Algae and Antiviral Drugs Containing the Same as Active Ingredient. European Patent.

[83] Fabra M.J., Falcó I., Randazzo W., Sánchez G., López-Rubio A. (2018). Antiviral and antioxidant properties of active alginate edible films containing phenolic extracts. Food Hydrocoll..

[84] Serrano-Aroca Á., Ferrandis-Montesinos M., Wang R. (2021). Antiviral Properties of Alginate-Based Biomaterials: Promising Antiviral Agents against SARS-CoV-2. ACS Appl. Bio Mater..

[85] Sano Y. (1999). Antiviral activity of alginate against infection by tobacco mosaic virus. Carbohydr. Polym..

[86] Cano-Vicent A., Hashimoto R., Takayama K., Serrano-Aroca Á. (2022). Biocompatible Films of Calcium Alginate Inactivate Enveloped Viruses such as SARS-CoV-2. Polymers.

[87] Vera J., Castro J., Gonzalez A., Moenne A. (2011). Seaweed polysaccharides and derived oligosaccharides stimulate defense responses and protection against pathogens in plants. Mar. Drugs.

[88] Qin Y. (2008). Alginate fibres: An overview of the production processes and applications in wound management. Polym. Int..

[89] Gao Y., Zhang L., Jiao W. (2019). Marine glycan-derived therapeutics in China. Progress in Molecular Biology and Translational Science.

[90] Szekalska M., Puciłowska A., Szymańska E., Ciosek P., Winnicka K. (2016). Alginate: Current Use and Future Perspectives in Pharmaceutical and Biomedical Applications. Int. J. Polym. Sci..

[91] Gaikwad M., Pawar Y., Nagle V., Santanu D. (2020). Marine Red Alga *Porphyridium* sp. as a Source of Sulfated Polysaccharides (SPs) for Combating against COVID-19. Preprints.

[92] Jabeen M., Dutot M., Fagon R., Verrier B., Monge C. (2021). Seaweed sulfated polysaccharides against respiratory viral infections. Pharmaceutics.

[93] Krylova N.V., Kravchenko A.O., Iunikhina O.V., Pott A.B., Likhatskaya G.N., Volod’ko A.V., Zaporozhets T.S., Shchelkanov M.Y., Yermak I.M. (2022). Influence of the Structural Features of Carrageenans from Red Algae of the Far Eastern Seas on Their Antiviral Properties. Mar. Drugs.

[94] McCandless E.L., Craigie J.S. (1979). Sulfated Polysaccharides in Red and Brown Algae. Annu. Rev. Plant Physiol..

[95] Therkelsen G.H. (2012). Carrageenan. Industrial Gums: Polysaccharides and Their Derivatives.

[96] Zhong H., Gao X., Cheng C., Liu C., Wang Q., Han X. (2020). The Structural Characteristics of Seaweed Polysaccharides and Their Application in Gel Drug Delivery Systems. Mar. Drugs.

[97] Necas J., Bartosikova L. (2013). Carrageenan: A review. Vet. Med..

[98] Carlucci M.J., Ciancia M., Matulewicz M.C., Cerezo A.S., Damonte E.B. (1999). Antiherpetic activity and mode of action of natural carrageenans of diverse structural types. Antivir. Res..

[99] Girond S., Crance J.M., Van Cuyck-Gandre H., Renaudet J., Deloince R. (1991). Antiviral activity of carrageenan on hepatitis A virus replication in cell culture. Res. Virol..

[100] Talarico L.B., Pujol C.A., Zibetti R.G.M., Faría P.C.S., Noseda M.D., Duarte M.E.R., Damonte E.B. (2005). The antiviral activity of sulfated polysaccharides against dengue virus is dependent on virus serotype and host cell. Antivir. Res..

[101] Buck C.B., Thompson C.D., Roberts J.N., Müller M., Lowy D.R., Schiller J.T. (2006). Carrageenan is a potent inhibitor of papillomavirus infection. PLoS Pathog..

[102] Grassauer A., Weinmuellner R., Meier C., Pretsch A., Prieschl-Grassauer E., Unger H. (2008). Iota-Carrageenan is a potent inhibitor of rhinovirus infection. Virol. J..

[103] Zhou G., Sun Y.P., Xin H., Zhang Y., Li Z., Xu Z. (2004). In vivo antitumor and immunomodulation activities of different molecular weight lambda-carrageenans from *Chondrus ocellatus*. Pharmacol. Res..

[104] Yuan H., Song J., Li X., Li N., Dai J. (2006). Immunomodulation and antitumor activity of κ-carrageenan oligosaccharides. Cancer Lett..

[105] Besednova N.N., Andryukov B.G., Zaporozhets T.S., Kryzhanovsky S.P., Fedyanina L.N., Kuznetsova T.A., Zvyagintseva T.N., Shchelkanov M.Y. (2021). Antiviral effects of polyphenols from marine algae. Biomedicines.

[106] Wink M. (2020). Potential of DNA intercalating alkaloids and other plant secondary metabolites against SARS-CoV-2 causing COVID-19. Diversity.

[107] Wink M. (2015). Modes of Action of Herbal Medicines and Plant Secondary Metabolites. Medicines.

[108] Venkatesan J., Keekan K.K., Anil S., Bhatnagar I., Kim S.K. (2018). Phlorotannins. Encycl. Food Chem..

[109] Yang H., Zeng M., Dong S., Liu Z., Li R. (2010). Anti-proliferative activity of phlorotannin extracts from brown algae *Laminaria japonica* Aresch. Chinese J. Oceanol. Limnol..

[110] Cotas J., Leandro A., Monteiro P., Pacheco D., Figueirinha A., Goncąlves A.M.M., Da Silva G.J., Pereira L. (2020). Seaweed phenolics: From extraction to applications. Mar. Drugs.

[111] Freile-Pelegrín Y., Robledo D. (2013). Bioactive Phenolic Compounds from Algae. Bioactive Compounds from Marine Foods: Plant and Animal Sources.

[112] Rengasamy K.R.R., Aderogba M.A., Amoo S.O., Stirk W.A., Van Staden J. (2013). Potential antiradical and alpha-glucosidase inhibitors from *Ecklonia maxima* (Osbeck) Papenfuss. Food Chem..

[113] Kwon H.-J., Ryu Y.B., Kim Y.-M., Song N., Kim C.Y., Rho M.-C., Jeong J.-H., Cho K.-O., Lee W.S., Park S.-J. (2013). In vitro antiviral activity of phlorotannins isolated from *Ecklonia cava* against porcine epidemic diarrhea coronavirus infection and hemagglutination. Bioorg. Med. Chem..

[114] Park J.Y., Yuk H.J., Ryu H.W., Lim S.H., Kim K.S., Park K.H., Ryu Y.B., Lee W.S. (2017). Evaluation of polyphenols from *Broussonetia papyrifera* as coronavirus protease inhibitors. J. Enzyme Inhib. Med. Chem..

[115] Hayashi K., Lee J.-B., Nakano T., Hayashi T. (2013). Anti-influenza A virus characteristics of a fucoidan from sporophyll of *Undaria pinnatifida* in mice with normal and compromised immunity. Microbes Infect..

[116] Hashem A.M., Flaman A.S., Farnsworth A., Brown E.G., Van Domselaar G., He R., Li X. (2009). Aurintricarboxylic Acid Is a Potent Inhibitor of Influenza A and B Virus Neuraminidases. PloS ONE.

[117] Richards C., Williams N.A., Fitton J.H., Stringer D.N., Karpiniec S.S., Park A.Y. (2020). Oral fucoidan attenuates lung pathology and clinical signs in a severe influenza a mouse model. Mar. Drugs.

[118] Kim M., Han J., Kim S., Soo H., Ghil W., Jin S., Kang P., Lee C. (2012). In vitro inhibition of influenza A virus infection by marine microalga-derived sulfated polysaccharide p-KG03. Antivir. Res..

[119] Ryu Y.B., Jeong H.J., Yoon S.Y., Park J.-Y., Kim Y.M., Park S.-J., Rho M.-C., Kim S.-J., Lee W.S. (2011). Influenza Virus Neuraminidase Inhibitory Activity of Phlorotannins from the Edible Brown Alga *Ecklonia cava*. J. Agric. Food Chem..

[120] Cho H.M., Doan T.P., Quy Ha T.K., Kim H.W., Lee B.W., Tung Pham H.T., Cho T.O., Oh W.K. (2019). Dereplication by High-Performance Liquid Chromatography (HPLC) with Quadrupole-Time-of-Flight Mass Spectroscopy (qTOF-MS) and Antiviral Activities of Phlorotannins from *Ecklonia cava*. Mar. Drugs.

[121] Yu G., Li M., Wang W., Liu X., Zhao X., Lv Y., Li G., Jiao G., Zhao X. (2012). Structure and anti-influenza A (H1N1) virus activity of three polysaccharides from *Eucheuma denticulatum*. J. Ocean Univ. China.

[122] Tang F., Chen F., Li F. (2013). Preparation and potential in vivo anti-influenza virus activity of low molecular-weight κ-carrageenans and their derivatives. J. Appl. Polym. Sci..

[123] Morokutti-Kurz M., König-Schuster M., Koller C., Graf C., Graf P., Kirchoff N., Reutterer B., Seifert J.-M., Unger H., Grassauer A. (2015). The Intranasal Application of Zanamivir and Carrageenan Is Synergistically Active against Influenza a Virus in the Murine Model. PLoS ONE.

[124] Leibbrandt A., Meier C., König-Schuster M., Weinmüllner R., Kalthoff D., Pflugfelder B., Graf P., Frank-Gehrke B., Beer M., Fazekas T. (2010). Iota-carrageenan is a potent inhibitor of influenza a virus infection. PLoS ONE.

[125] Shao Q., Guo Q., Xu W.P., Li Z., Zhao T.T. (2015). Specific inhibitory effect of κ-carrageenan polysaccharide on swine pandemic 2009 H1N1 influenza virus. PLoS ONE.

[126] Jang Y., Shin H., Lee M.K., Kwon O.S., Shin J.S., Kim Y., Kim C.W., Lee H.-R., Kim M. (2021). Antiviral activity of lambda-carrageenan against influenza viruses and severe acute respiratory syndrome coronavirus 2. Sci. Rep..

[127] Synytsya A., Bleha R., Synytsya A., Pohl R., Hayashi K., Yoshinaga K., Nakano T., Hayashi T. (2014). Mekabu fucoidan: Structural complexity and defensive effects against avian influenza A viruses. Carbohydr. Polym..

[128] Thuy T.T.T., Ly B.M., Van T.T.T., Van Quang N., Tu H.C., Zheng Y., Seguin-Devaux C., Mi B., Ai U. (2015). Anti-HIV activity of fucoidans from three brown seaweed species. Carbohydr. Polym..

[129] Sanniyasi E., Venkatasubramanian G., Anbalagan M.M., Raj P.P., Gopal R.K. (2019). In vitro anti-HIV-1 activity of the bioactive compound extracted and purified from two different marine macroalgae (seaweeds) (*Dictyota bartayesiana* J.V. Lamouroux and *Turbinaria decurrens* Bory). Sci. Rep..

[130] Prokofjeva M.M., Imbs T.I., Shevchenko N.M., Spirin P.V., Horn S., Fehse B., Zvyagintseva T.N., Prassolov V.S. (2013). Fucoidans as potential inhibitors of HIV-1. Mar. Drugs.

[131] Trinchero J., Ponce N.M.A., Córdoba O.L., Flores M.L., Pampuro S., Stortz C.A., Salomón H., Turk G. (2009). Antiretroviral Activity of Fucoidans Extracted from the Brown Seaweed *Adenocystis utricularis*. Phyther. Res..

[132] Santos J.P., Torres P.B., dos Santos D.Y.A.C., Motta L.B., Chow F. (2019). Seasonal effects on antioxidant and anti-HIV activities of Brazilian seaweeds. J. Appl. Phycol..

[133] Harb T.B., Chow F. (2022). Anti-HIV activity of methanolic and aqueous extracts of fifteen materials of beach-cast macroalgae: Valorization of underused waste biomass. Appl. Phycol..

[134] Polo L.K., Chow F. (2022). Variation of antioxidant capacity and antiviral activity of the brown seaweed *Sargassum filipendula* (*Fucales*, *Ochrophyta*) under UV radiation treatments. Appl. Phycol..

[135] Artan M., Li Y., Karadeniz F., Lee S.H., Kim M.M., Kim S.K. (2008). Anti-HIV-1 activity of phloroglucinol derivative, 6,6′-bieckol, from *Ecklonia cava*. Bioorganic Med. Chem..

[136] Ahn M.J., Yoon K.D., Min S.Y., Lee J.S., Kim J.H., Kim T.G., Kim S.H., Kim N.G., Huh H., Kim J. (2004). Inhibition of HIV-1 reverse transcriptase and protease by phlorotannins from the brown alga *Ecklonia cava*. Biol. Pharm. Bull..

[137] Santos M.G.M., Lagrota M.H.C., Miranda M.M.F.S., Yoneshigue-Valentin Y., Wigg M.D. (1999). A screening for the antiviral effect of extracts from Brazilian marine algae against acyclovir resistant herpes simplex virus type 1. Bot. Mar..

[138] Wang H., Ooi E.V., Ang P.O. (2008). Antiviral activities of extracts from Hong Kong seaweeds. J. Zhejiang Univ. Sci. B.

[139] Hayashi K., Nakano T., Hashimoto M., Kanekiyo K., Hayashi T. (2008). Defensive effects of a fucoidan from brown alga *Undaria pinnatifida* against herpes simplex virus infection. Int. Immunopharmacol..

[140] Bouhlal R., Haslin C., Chermann J.C., Colliec-Jouault S., Sinquin C., Simon G., Cerantola S., Riadi H., Bourgougnon N. (2011). Antiviral activities of sulfated polysaccharides isolated from *Sphaerococcus coronopifolius* (*Rhodophytha*, *Gigartinales*) and *Boergeseniella thuyoides* (*Rhodophyta*, *Ceramiales*). Mar. Drugs.

[141] Boulho R., Marty C., Freile-Pelegrín Y., Robledo D., Bourgougnon N., Bedoux G. (2017). Antiherpetic (HSV-1) activity of carrageenans from the red seaweed *Solieria chordalis* (*Rhodophyta*, *Gigartinales*) extracted by microwave-assisted extraction (MAE). J. Appl. Phycol..

[142] Bedoux G., Caamal-Fuentes E., Boulho R., Marty C., Bourgougnon N., Freile-Pelegrín Y., Robledo D. (2017). Antiviral and cytotoxic activities of polysaccharides extracted from four tropical seaweed species. Nat. Prod. Commun..

[143] Kulshreshtha G., Burlot A.S., Marty C., Critchley A., Hafting J., Bedoux G., Bourgougnon N., Prithiviraj B. (2015). Enzyme-assisted extraction of bioactive material from *Chondrus crispus* and *Codium fragile* and its effect on Herpes simplex virus (HSV-1). Mar. Drugs.

[144] Soares A.R., Robaina M.C.S., Mendes G.S., Silva T.S.L., Gestinari L.M.S., Pamplona O.S., Yoneshigue-Valentin Y., Kaiser C.R., Romanos M.T.V. (2012). Antiviral activity of extracts from Brazilian seaweeds against herpes simplex virus. Braz. J. Pharmacogn..

[145] Pujol C.A., Errea M.I., Matulewicz M.C., Damonte E.B. (1996). Antiherpetic activity of S1, an algal derived sulphated galactan. Phyther. Res..

[146] Rhimou B., Hassane R., Nathalie B. (2010). Antiviral activity of the extracts of Rhodophyceae from Morocco. Afr. J. Biotechnol..

[147] Pliego-Cortés H., Hardouin K., Bedoux G., Marty C., Cérantola S., Freile-Pelegrín Y., Robledo D., Bourgougnon N. (2022). Sulfated Polysaccharides from Seaweed Strandings as Renewable Source for Potential Antivirals against *Herpes simplex* Virus 1. Mar. Drugs.

[148] Talarico L.B., Zibetti R.G.M., Faria P.C.S., Scolaro L.A., Duarte M.E.R., Noseda M.D., Pujol C.A., Damonte E.B. (2004). Anti-herpes simplex virus activity of sulfated galactans from the red seaweeds *Gymnogongrus griffithsiae* and *Cryptonemia crenulata*. Int. J. Biol. Macromol..

[149] Sun Q.L., Li Y., Ni L.Q., Li Y.X., Cui Y.S., Jiang S.L., Xie E.Y., Du J., Deng F., Dong C.X. (2020). Structural characterization and antiviral activity of two fucoidans from the brown algae *Sargassum henslowianum*. Carbohydr. Polym..

[150] Mendes S., Bravin I.C., Yokoya N.S., Villela M.T. (2012). Anti-HSV activity of *Hypnea musciformis* cultured with different phytohormones. Braz. J. Pharmacogn..

[151] Carlucci M.J., Pujol C.A., Ciancia M., Noseda M.D., Matulewicz M.C., Damonte E.B., Cerezo A.S. (1997). Antiherpetic and anticoagulant properties of carrageenans from the red seaweed *Gigartina skottsbergii* and their cyclized derivatives: Correlation between structure and biological activity. Int. J. Biol. Macromol..

[152] Dhanasezhian A., Srivani S., Govindaraju K., Parija P., Sasikala S., Ramesh Kumar M.R. (2019). Anti-herpes simplex virus (HSV-1 and HSV-2) activity of biogenic gold and silver nanoparticles using seaweed *Sargassum wightii*. Indian J. Geo-Mar. Sci..

[153] Vissani M.A., Galdo Novo S., Ciancia M., Zabal O., Thiry E., Bratanich A., Barrandeguy M. (2016). Effects of lambda-carrageenan on equid herpesvirus 3 in vitro. J. Equine Vet. Sci..

[154] Carlucci M.J., Scolaro L.A., Noseda M.D., Cerezo A.S., Damonte E.B. (2004). Protective effect of a natural carrageenan on genital *Herpes simplex* virus infection in mice. Antivir. Res..

[155] Nixon B., Stefanidou M., Mesquita P.M.M., Fakioglu E., Segarra T., Rohan L., Halford W., Palmer K.E., Herold B.C. (2013). Griffithsin Protects Mice from Genital Herpes by Preventing Cell-to-Cell Spread. J. Virol..

[156] Derby N., Lal M., Aravantinou M., Kizima L., Barnable P., Rodriguez A., Lai M., Wesenberg A., Ugaonkar S., Levendosky K. (2018). Griffithsin carrageenan fast dissolving inserts prevent SHIV HSV-2 and HPV infections in vivo. Nat. Commun..

[157] Levendosky K., Mizenina O., Martinelli E., Jean-Pierre N., Kizima L., Rodriguez A., Kleinbeck K., Bonnaire T., Robbiani M., Zydowsky T.M. (2015). Griffithsin and carrageenan combination to target herpes simplex virus 2 and human papillomavirus. Antimicrob. Agents Chemother..

[158] Tyo K.M., Lasnik A.B., Zhang L., Mahmoud M., Jenson A.B., Fuqua J.L., Palmer K.E., Steinbach-Rankins J.M. (2020). Sustained-release Griffithsin nanoparticle-fiber composites against HIV-1 and HSV-2 infections. J. Control. Release.

[159] Kwon P.S., Oh H., Kwon S.-J., Jin W., Zhang F., Fraser K., Hong J.J., Linhardt R.J., Dordick J.S. (2020). Sulfated polysaccharides effectively inhibit SARS-CoV-2 in vitro. Cell Discov..

[160] Song S., Peng H., Wang Q., Liu Z., Dong X., Wen C., Ai C., Zhang Y., Wang Z., Zhu B. (2020). Inhibitory activities of marine sulfated polysaccharides against SARS-CoV-2. Food Funct..

[161] Morokutti-Kurz M., Fröba M., Graf P., Große M., Grassauer A., Auth J., Schubert U., Prieschl-Grassauer E. (2021). Iota-carrageenan neutralizes SARS-CoV-2 and inhibits viral replication in vitro. PLoS ONE.

[162] Varese A., Paletta A., Ceballos A., Palacios C.A., Figueroa J.M., Dugour A.V. (2021). Iota-Carrageenan Prevents the Replication of SARS-CoV-2 in a Human Respiratory Epithelium Cell Line In Vitro. Front. Virol..

[163] Fröba M., Große M., Setz C., Rauch P., Auth J., Spanaus L., Münch J., Ruetalo N., Schindler M., Morokutti-Kurz M. (2021). Iota-carrageenan inhibits replication of sars-cov-2 and the respective variants of concern alpha, beta, gamma and delta. Int. J. Mol. Sci..

[164] Koenighofer M., Lion T., Bodenteich A., Prieschl-Grassauer E., Grassauer A., Unger H., Mueller C.A., Fazekas T. (2014). Carrageenan nasal spray in virus confirmed common cold: Individual patient data analysis of two randomized controlled trials. Multidiscip. Respir. Med..

[165] Negishi H., Mori M., Mori H., Yamori Y. (2013). Supplementation of elderly Japanese men and women with fucoidan from seaweed increases immune responses to seasonal influenza vaccination. J. Nutr..

[166] Eccles R., Meier C., Jawad M., Weinmüllner R., Grassauer A., Prieschl-Grassauer E. (2010). Efficacy and safety of an antiviral Iota-Carrageenan nasal spray: A randomized, double-blind, placebo-controlled exploratory study in volunteers with early symptoms of the common cold. Respir. Res..

[167] Ludwig M., Enzenhofer E., Schneider S., Rauch M., Bodenteich A., Neumann K., Prieschl-Grassauer E., Grassauer A., Lion T., Mueller C.A. (2013). Efficacy of a Carrageenan nasal spray in patients with common cold: A randomized controlled trial. Respir. Res..

[168] Tsubura S., Suzuki A. (2018). Case Report Using 4% Fucoidan Cream for Recurrent Oral Herpes Labialis: Patient Symptoms Markedly Improved in Terms of Time to Healing and Time to Loss of Discomfort. Dent.-Open J..

[169] Figueroa J.M., Lombardo M.E., Dogliotti A., Flynn L.P., Giugliano R., Simonelli G., Valentini R., Ramos A., Romano P., Marcote M. (2021). Efficacy of a nasal spray containing iota-carrageenan in the postexposure prophylaxis of COVID-19 in hospital personnel dedicated to patients care with COVID-19 disease. Int. J. Gen. Med..

[170] Álvarez-Viñas M., Souto S., Flórez-Fernández N., Torres M.D., Bandín I., Domínguez H. (2021). Antiviral activity of carrageenans and processing implications. Mar. Drugs.

[171] Wang W., Zhang P., Hao C., Zhang X.-E., Cui Z.-Q., Guan H.-S. (2011). In vitro inhibitory effect of carrageenan oligosaccharide on influenza A H1N1 virus. Antivir. Res..

[172] De Clercq E. (2005). Antiviral drug discovery and development: Where chemistry meets with biomedicine. Antivir. Res..

[173] Skoler-Karpoff S., Ramjee G., Ahmed K., Altini L., Plagianos M.G., Friedland B., Govender S., De Kock A., Cassim N., Palanee T. (2008). Efficacy of Carraguard for prevention of HIV infection in women in South Africa: A randomised, double-blind, placebo-controlled trial. Lancet.

[174] Lynch G., Low L., Li S., Sloane A., Adams S., Parish C., Kemp B., Cunningham A.L. (1994). Sulfated polyanions prevent HIV infection of lymphocytes by disruption of the CD4-gp120 interaction, but do not inhibit monocyte infection. J. Leukoc. Biol..

[175] Yamada T., Ogamo A., Saito T., Watanabe J., Uchiyama H., Nakagawa Y. (1997). Preparation and anti-HIV activity of low-molecular-weight carrageenans and their sulfated derivatives. Carbohydr. Polym..

[176] Emau P., Tian B., O’keefe B.R., Mori T., McMahon J.B., Palmer K.E., Jiang Y., Bekele G., Tsai C.C. (2007). Griffithsin, a potent HIV entry inhibitor, is an excellent candidate for anti-HIV microbicide. J. Med. Primatol..

[177] O’Brien J.J., Campoli-Richards D.M. (1989). Acyclovir: An Updated Review of Its Antiviral Activity, Pharmacokinetic Properties and Therapeutic Efficacy. Drugs.

[178] Corey L., Benedetti J.K., Critchlow C.W., Remington M.R., Winter C.A., Fahnlander A.L., Smith K., Salter D.L., Keeney R.E., Davis L.G. (1982). Double-blind controlled trial of topical acyclovir in genital herpes simplex virus infections. Am. J. Med..

[179] Galdiero S., Falanga A., Vitiello M., Cantisani M., Marra V., Galdiero M. (2011). Silver Nanoparticles as Potential Antiviral Agents. Molecules.

[180] Russo L., Galdiero S., Galdiero M. (2013). Antiviral activity of mycosynthesized silver nanoparticles against herpes simplex virus and human parainfluenza virus type 3. Int. J. Nanomed..

[181] Munazza F., Zaidi N.-S.S., Amraiz D., Afzal F. (2016). In Vitro Antiviral Activity of *Cinnamomum cassia* and Its Nanoparticles Against H7N3 Influenza A Virus. J. Microbiol. Biotechnol..

[182] Broglie J.J., Alston B., Yang C., Ma L., Adcock A.F. (2015). Antiviral Activity of Gold / Copper Sulfide Core/Shell Nanoparticles against Human Norovirus Virus-Like Particles. PLoS ONE.

[183] Elechiguerra J.L., Burt J.L., Morones J.R., Camacho-Bragado A., Gao X., Lara H.H., Yacaman M.J. (2005). Interaction of silver nanoparticles with HIV-1. J. Nanobiotechnol..

[184] Maneesh A., Chakraborty K., Makkar F. (2017). Pharmacological activities of brown seaweed *Sargassum wightii* (family *Sargassaceae*) using different in vitro models. Int. J. Food Prop..

[185] Pereira L., Critchley A.T. (2020). The COVID 19 novel coronavirus pandemic 2020: Seaweeds to the rescue? Why does substantial, supporting research about the antiviral properties of seaweed polysaccharides seem to go unrecognized by the pharmaceutical community in these desperate times?. J. Appl. Phycol..

[186] Li H., Liu L., Zhang D., Xu J., Dai H., Tang N., Su X., Cao B. (2020). Hypothesis SARS-CoV-2 and viral sepsis: Observations and hypotheses. Lancet.

[187] Yang Y., Tang H. (2016). Aberrant coagulation causes a hyper-inflammatory response in severe influenza pneumonia. Cell. Mol. Immunol..

[188] Zuo T., Zhang F., Lui G.C.Y., Yeoh Y.K., Li A.Y.L., Zhan H., Wan Y., Chung A.C.K., Cheung C.P., Chen N. (2020). Alterations in Gut Microbiota of Patients with COVID-19 during Time of Hospitalization. Gastroenterology.

[189] He Y., Wang J., Li F., Shi Y. (2020). Main Clinical Features of COVID-19 and Potential Prognostic and Therapeutic Value of the Microbiota in SARS-CoV-2 Infections. Front. Microbiol..

[190] Pradhan B., Nayak R., Patra S., Jit B.P., Ragusa A., Jena M. (2020). Bioactive Metabolites from Marine Algae as Potent Pharmacophores against Oxidative Stress-Associated Human Diseases: A Comprehensive Review. Molecules.

[191] Fazekas T., Eickhoff P., Pruckner N., Vollnhofer G., Fischmeister G., Diakos C., Rauch M., Verdianz M., Zoubek A., Gadner H. (2012). Lessons learned from a double-blind randomised placebo-controlled study with a iota-carrageenan nasal spray as medical device in children with acute symptoms of common cold. BMC Complement. Altern. Med..

[192] Graf C., Bernkop-Schnürch A., Egyed A., Koller C., Prieschl-Grassauer E., Morokutti-Kurz M. (2018). Development of a nasal spray containing xylometazoline hydrochloride and iota-carrageenan for the symptomatic relief of nasal congestion caused by rhinitis and sinusitis. Int. J. Gen. Med..

